# Host lipid droplets: An important source of lipids salvaged by the intracellular parasite *Toxoplasma gondii*

**DOI:** 10.1371/journal.ppat.1006362

**Published:** 2017-06-01

**Authors:** Sabrina J. Nolan, Julia D. Romano, Isabelle Coppens

**Affiliations:** Department of Molecular Microbiology and Immunology, Johns Hopkins University Bloomberg School of Public Health, Baltimore, Maryland, United States of America; Wellcome Trust Sanger Institute, UNITED KINGDOM

## Abstract

*Toxoplasma* is an obligate intracellular parasite that replicates in mammalian cells within a parasitophorous vacuole (PV) that does not fuse with any host organelles. One mechanism developed by the parasite for nutrient acquisition is the attraction of host organelles to the PV. Here, we examined the exploitation of host lipid droplets (LD), ubiquitous fat storage organelles, by *Toxoplasma*. We show that *Toxoplasma* replication is reduced in host cells that are depleted of LD, or impaired in TAG lipolysis or fatty acid catabolism. In infected cells, the number of host LD and the expression of host LD-associated genes (*ADRP*, *DGAT2*), progressively increase until the onset of parasite replication. Throughout infection, the PV are surrounded by host LD. *Toxoplasma* is capable of accessing lipids stored in host LD and incorporates these lipids into its own membranes and LD. Exogenous addition of oleic acid stimulates LD biogenesis in the host cell and results in the overaccumulation of neutral lipids in very large LD inside the parasite. To access LD-derived lipids, *Toxoplasma* intercepts and internalizes within the PV host LD, some of which remaining associated with Rab7, which become wrapped by an intravacuolar network of membranes (IVN). Mutant parasites impaired in IVN formation display diminished capacity of lipid uptake from host LD. Moreover, parasites lacking an IVN-localized phospholipase A2 are less proficient in salvaging lipids from host LD in the PV, suggesting a major contribution of the IVN for host LD processing in the PV and, thus lipid content release. Interestingly, gavage of parasites with lipids unveils, for the first time, the presence in *Toxoplasma* of endocytic-like structures containing lipidic material originating from the PV lumen. This study highlights the reliance of *Toxoplasma* on host LD for its intracellular development and the parasite’s capability in scavenging neutral lipids from host LD.

## Author summary

*Toxoplasma* is an obligate intracellular pathogen that multiplies in mammalian cells within a specialized compartment, named the parasitophorous vacuole (PV). While the vacuole does not fuse with host organelles, the parasite scavenges nutrients, including lipids, from these compartments. Present in all mammalian cells, lipid droplets (LD) are dynamic structures that store neutral lipids. Whether *Toxoplasma* targets host LD for their nutritional content remains to be investigated. We demonstrate that the parasite relies on host LD lipids and their lipolytic enzymatic activities to grow. *Toxoplasma* salvages lipids from host LD, which surround the PV and, at least partially, accesses these lipids by intercepting and engulfing within the PV host Rab7-associated LD. In the PV lumen, a parasite lipase releases lipids from host LD, thus making them available to the parasite. Exogenous addition of fatty acids stimulates host LD biogenesis and results in the accumulation of enlarged LD containing neutral lipids in *Toxoplasma*. This study highlights the ability of *Toxoplasma* to scavenge and store lipids from host LD. Interestingly, exposure of *Toxoplasma* to excess lipids reveals, for the first time, coated invaginations of the parasite’s plasma membrane and cytoplasmic vesicles containing lipids originating from the PV lumen, potentially involved in endocytosis.

## Introduction

In mammalian cells, lipid droplets (LD) are cytoplasmic structures containing a diverse array of lipids and proteins. LD consist of an organic core, comprising neutral lipids (mostly triacylglycerols (TAG) and cholesteryl esters (CE) with mixed fatty acid composition) bounded by a monolayer of phospholipids [1]. Inserted onto the outer phospholipid monolayer are structural proteins (e.g., proteins of the perilipin family, such as Adipose differentiation-related protein (ADRP) or adipophilin), lipid biosynthetic enzymes (e.g., acyl-CoA:diacylglycerol acyltransferase 2 (DGAT2), and acyl-CoA synthetase), lipolytic enzymes (e.g., adipose tissue triacylglycerol lipase (ATGL) and membrane-trafficking proteins (e.g., Rab7, Rab18 and ARF1) [2–4]. LD display canonical lipid-related functions ranging from energy storage to lipid homeostasis. In addition, these structures are engaged in various cellular processes, depending on cell type and activation conditions, such as cell signaling during inflammation, innate immunity, RNA metabolism, cytoskeletal organization, nuclear transcription and histone modulation [5–7]. Pertinent to the role of LD in immune responses, LD produce inflammation mediators (e.g., prostaglandins and leukotrienes), regulate the MHC-I antigen presentation pathway, and are assembling platforms for effectors involved in interferon response [8].

Unsurprisingly, several pathogens have evolved to take advantage of host LD to favor their own survival: they target host LD for progeny assembly, or as part of an anti-immunity strategy [8–12]. Prominent pathogen-mediated changes to host LD include inducing LD formation, altering LD ultrastructure, modifying LD lipid and protein composition, relocating LD to the site of pathogen replication, and transferring LD content to the pathogen’s intracellular compartment. Some intracellular pathogens activate host intracellular signaling pathways, leading to enhanced LD formation. For instance, *Trypanosoma cruzi* and *Mycobacterium tuberculosis* trigger LD biogenesis in macrophages through a Toll-like receptor-2-dependent mechanism [13, 14]. Additionally, *Chlamydia trachomatis* and *M*. *tuberculosis* sequester host LD in their phagosome-like compartment [15–17]. Thus, it has been proposed that host LD-stored lipids could serve as nutrients, though the transfer of lipids derived from LD to the phagosome or bacterial cell has never been experimentally demonstrated.

The protozoan parasite, *Toxoplasma gondii* causes life-threatening diseases, such as toxoplasmic encephalitis, in immunocompromised individuals [18]. *T*. *gondii* infects a large variety of mammalian cells wherein it resides in a self-made parasitophorous vacuole (PV). The production of infectious progeny and persistence of the parasite in its host importantly rely on lipid metabolic activities. *Toxoplasma* acquires the necessary lipids through intricate and complex networks of synthetic and salvage pathways [19, 20]. For instance, we previously showed that *T*. *gondii* is auxotrophic for low-density lipoprotein-derived cholesterol and retrieves this lipid from mammalian endocytic organelles [21]. Once internalized by the parasite, cholesterol can be esterified for storage in cytosolic LD by two acyl-CoA:cholesterol acyltransferases (ACAT) enzymes [22, 23]. The parasite also intercepts host cell Golgi-derived Rab14, Rab30 and Rab43 vesicles to scavenge their sphingolipid content [24]. Thus, *T*. *gondii* largely exploits the host lipidome.

Recent studies on *Toxoplasma* infecting macrophages and skeletal muscle cells showed an increased number of LD in the host cytoplasm up to 48 h post-infection [25, 26]. This enhanced LD formation in *Toxoplasma*-infected cells correlates with reduced host microbicidal properties, such as an increased synthesis of PGE_2_, a potent inhibitor of Th1 type response, and a decreased production of nitric oxide. Aside from the augmented production of eicosanoids, the propagation of lipid-rich reservoirs may be beneficial for a parasite that forages for lipids from many sources in the host cell.

In this study, we explored the ability of *T*. *gondii* to exploit host LD for nutritional purposes. Our data reveal that host LD lipid content and lipolytic activities are important for the parasite’s development in mammalian cells. *Toxoplasma* infection has a pronounced impact on host cell neutral lipid homeostasis, through changes in host LD abundance, LD intracellular distribution and host neutral lipid syntheses. We also provide morphological evidence that *Toxoplasma* internalizes host LD into the PV and salvages their lipid content for incorporation into membranes or, in case of excess, storage in its own LD.

## Results

### During *Toxoplasma* infection, the number of host cell lipid droplets progressively increases then declines

**Fig 1:**
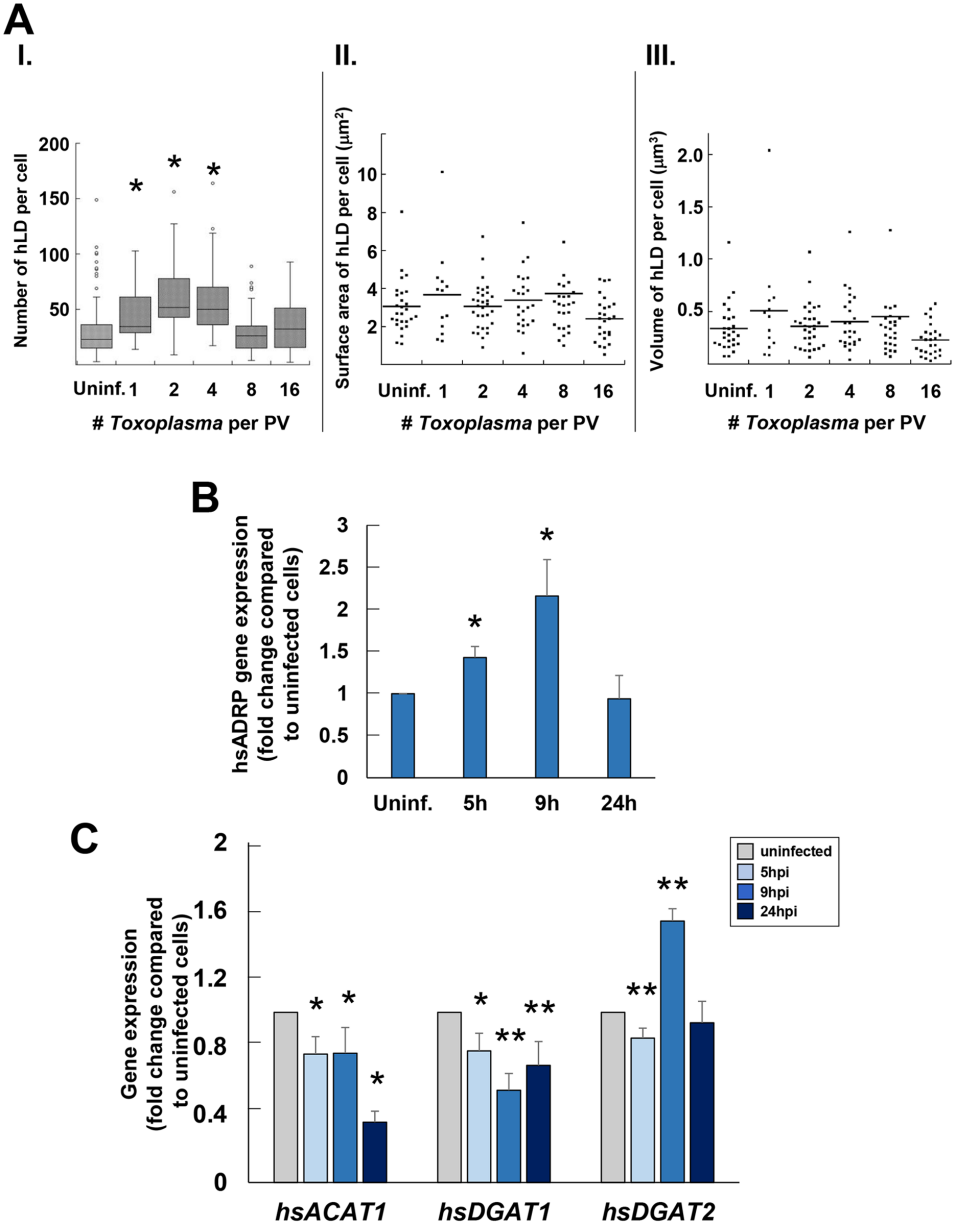
Influence of *Toxoplasma* infection on host LD number and composition. **A.** Properties of host LD in infected HFF. Determination, using Volocity software, of the host LD (hLD) number (in I), surface area (in II) and volume (inIII) throughout infection. Data were acquired from 2 independent experiments, and were categorized by the number of parasites per PV (> 30 PV counted per experiment for each sample). In panels II and III, the values of host LD surface area and volume, expressed in μm^2^ and μm^3^ respectively, represent the mean surface area and volume of host LD in individual cells, grouped together (n < 30 cells per replicate experiment), of which there were no statistical differences. Significant differences were observed for host LD number, which increased in cells containing PV with a single, 2 and 4 parasites, as compared to uninfected cells (*p <0.05). **B.** Human Adipose Differentiation-Related Protein (hsADRP) gene expression in infected HFF. Real-time PCR analysis of hsADRP transcripts throughout infection. Data are means ± SD of 3 separate assays performed in biological triplicates, showing significant increase of transcript levels of hsADRP at early time points of infection compared to uninfected cells (*p* <0.001) **C.** Human Acetyl-Coenzyme A Acetyltransferase 1 (hsACAT1), Diacylglycerol O-Acyltransferase 1 (hsDGAT1) and Diacylglycerol O-Acyltransferase 2 (hsDGAT2) gene expression in infected HFF. Real-time PCR analysis of *hsACAT1*, *hsDGAT1* and *hsDGAT2* throughout *Toxoplasma* infection. Data are means ± SD of 3 separate assays performed with biological triplicates, showing significant changes in transcript levels of the three genes early in infection compared to uninfected cells (*p <0.0003; **p <0.0001).

It has been shown that host LD biogenesis is stimulated in *Toxoplasma*-infected mouse macrophages and skeletal muscle cells 24 h and 48 h post-infection (p.i.) [25, 26]. To determine if a similar effect occurs in other cell types, human fibroblasts (HFF) and epithelial cells (HeLa) were infected *in vitro* with *Toxoplasma*, and LD biogenesis was monitored throughout infection. Infected HFF at various time points were stained with BODIPY 493/503, a lipophilic dye that specifically accumulates and fluoresces in LD, and the host LD number was assessed by fluorescence microscopy (Fig 1A, panel I). The number of mammalian LD steadily increased early in infection from a median of 23 LD in uninfected cells to 34 (1/PV), 51 (2/PV) and 50 (4/PV) in infected cells. However, once the PV contains 8 parasites, which corresponds to ~24 h p.i., the host LD number abruptly declined to approximately 26 per cell. The mean surface area and volume of mammalian LD per cell did not significantly differ between uninfected and infected HFF (Fig 1A, panels II and III). To validate the variation in host LD numbers during a *Toxoplasma* infection, we measured the transcriptional levels of the gene coding for ADRP, a protein associated with the LD surface [27] (Fig 1B). The expression of *hsADRP* transcripts increased by 1.5-fold at 5 h p.i., as compared to uninfected cells, and continued increasing up to 9 h p.i., then declined to levels similar to uninfected cells at 24 h p.i. The peak of *hsADRP* expression (~2-fold increase) at 9 h p.i. (Fig 1B), a time period when the majority of the PV contains two parasites, coincides then with the maximal number of host LD in infected cells (Fig 1A, panel I). This reflects a highly dynamic status of host LD upon *Toxoplasma* infection, with an increased number of LD, then a decline from 24 h p.i. until parasite egress.

### *Toxoplasma* infection results in transcriptional changes for host enzymes that produce neutral lipids

LD formation is closely linked to the biosynthesis of neutral lipids, e.g., cholesteryl esters (CE) and triacyglycerols (TAG) that are packaged in the LD core [28, 29]. In mammalian cells, CE and TAG are formed via distinct biosynthetic pathways located to the ER. The final step of CE biosynthesis is catalyzed by two ACAT enzymes that esterifies cholesterol: ACAT1 synthesizes CE destined for storage in cytoplasmic LD while ACAT2 forms CE for packaging into plasma lipoproteins, respectively [30, 31]. TAG formation is catalyzed by two DGAT enzymes that convert diacylglycerol (DAG) and acyl-CoA-activated fatty acid into TAG [32]. DGAT1 has a dual activity: it esterifies exogenous fatty acids taken up by cells and it acts in a recycling pathway involving the re-esterification of hydrolyzed TAG. DGAT2 is responsible for incorporating endogenously synthesized monounsaturated fatty acid into TAG, and is linked with fatty acid biosynthetic pathways, e.g., the stearoyl-CoA desaturase (SCD) pathway. Upon reaching a threshold in CE and TAG concentrations within the ER bilayer, the nascent LD detaches itself from the ER [7]. Located in the cytoplasmic leaflet of the ER, DGAT2 readily diffuses onto the nascent LD and eventually resides on mature LD where it continuously produces TAG [33]. As *Toxoplasma* infection results in changes in host LD number, we examined whether the production of CE or TAG is modified in infected cells by monitoring the transcriptional activities of host ACAT1, DGAT1 and DGAT2 at 5 h, 9 h and 24 h p.i. (Fig 1C). A significant down-regulation of *hsACAT1* expression throughout infection was observed with ~2.8-times fewer transcripts by 24 h p.i., suggesting a decrease in CE synthesis in host cells. Expression levels of *hsDGAT1* were also reduced in infected cells, reflecting a decrease in DAG esterification with exogenous fatty acids and/or decrease in TAG re-esterification via the recycling pathway. While *hsDGAT2* expression decreased slightly 5 h p.i., it increased significantly by 1.6-times 9 h p.i., as compared to uninfected cells. This increase in *hsDGAT2* expression correlates with the observed increase in host LD number (Fig 1A, panel I). As DGAT2 is in part located on host LD, this suggests a local increase in TAG production in these structures.

### Host lipid droplets cluster around the PV of *Toxoplasma*

**Fig 2:**
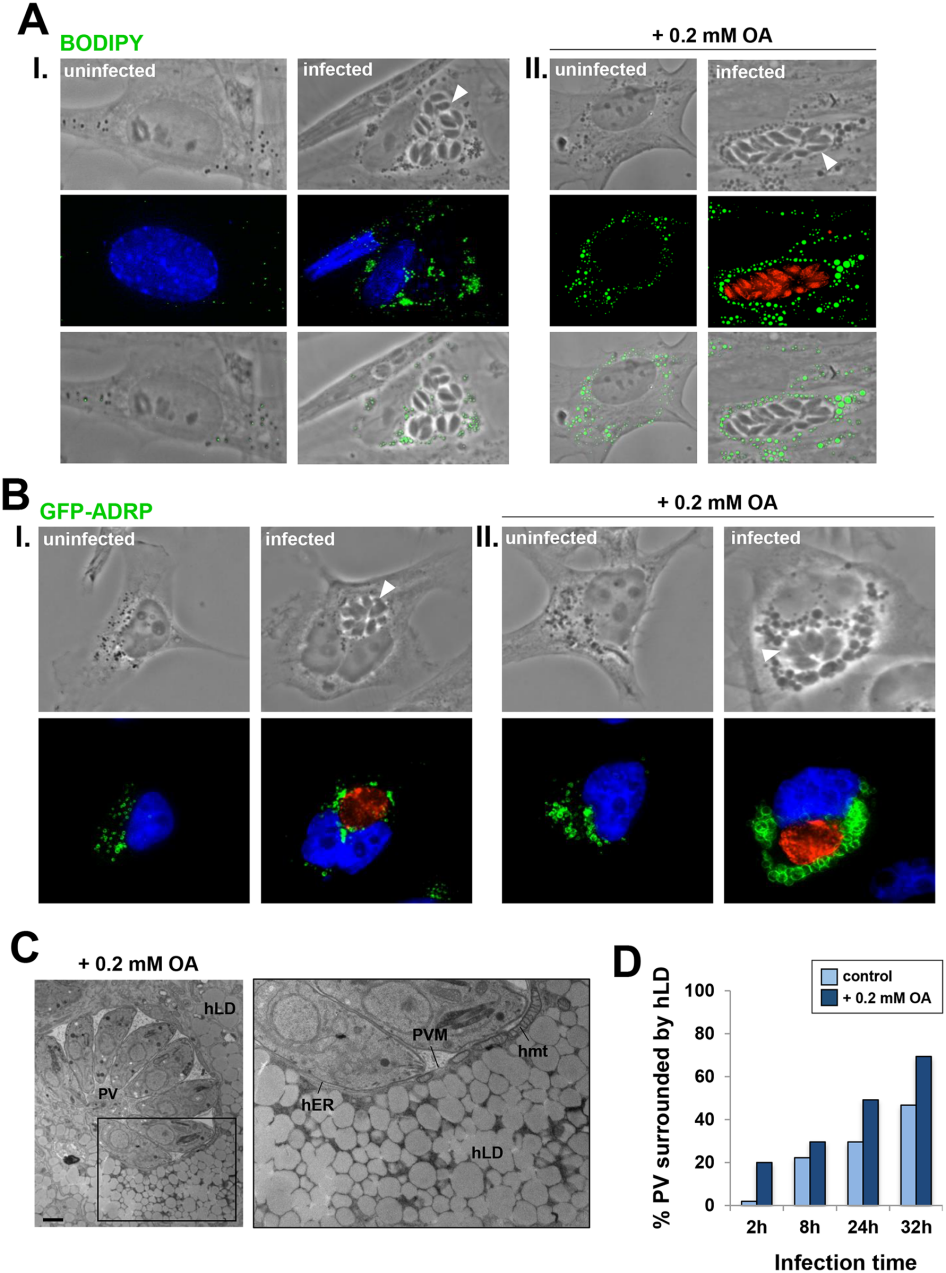
Influence of *Toxoplasma* infection on host LD distribution. **A.** Fluorescence microscopy of HFF infected with RFP-expressing *Toxoplasma* or WT for 24 h. Host LD were identified by staining with BODIPY 493/503 (green) and 4-,6-diamidino-2-phenylindole (DAPI, blue, nucleus). Uninfected and infected cells were incubated either under control conditions (corresponding to culture medium supplemented with 10% FBS that contains on average 3 μg/ml OA; in I) or with 0.2 mM OA added to the medium (corresponding to a ~20-fold excess of OA; in II). Images show host LD gathering around each PV, which was more pronounced upon OA addition. Arrowheads pinpoint PV. **B.** Fluorescence microscopy of GFP-ADRP-expressing HeLa cells infected with *Toxoplasma* for 24 h. The PV (arrowheads) were immunostained for GRA7 (red) in cells incubated with (in I) or without OA (in II). GFP-labeled host LD encircle the PV. In blue, DAPI. **C.** EM of HFF infected with *Toxoplasma* for 24 h and incubated with 0.2 mM OA during infection, confirming the amassing of host LD at the PV membrane (PVM). Host ER (hER) and mitochondria (hmt) remained associated with the vacuolar membrane (inset). Scale bar, 1 μm. **D.** Quantification of the percent of the PV population surrounded by host LD during infection, with or without exogenous 0.2 mM OA. GFP-ADRP-expressing HeLa cells were infected with *Toxoplasma* at the indicated times. PV were scored as LD-associated if > 70% of the total host LD population was grouped around the PV.

A hallmark of a *Toxoplasma* infection is the attraction of many host organelles to the PV [24, 34–37]. As several pathogens are able to recruit LD around their vacuoles [11], we inspected the spatial distribution of host LD in *Toxoplasma*-infected HFF or HeLa cells. Infected HFF were stained with BODIPY 493/503 to visualize host lipid droplets (Fig 2A, panel I). In uninfected cells, LD were dispersed throughout the cytosol, or in some instances were perinuclear. In contrast in infected cells, the vast majority of LD gathered around individual PV of *Toxoplasma* beginning early in infection, and this perivacuolar distribution of host LD was maintained throughout infection until parasite egress.

We next examined the host LD-PV interaction under conditions of enhanced LD formation. LD biogenesis can be induced in mammalian cells by adding oleic acid (OA) to culture medium (Fig 2A, panel II) or by overexpressing ADRP in cells [38] (Fig 2B). Upon exogenous addition of 0.2 mM OA (corresponding to ~20-fold excess OA), we observed a pronounced increase in host LD association with the PV in HFF, as compared to control conditions. In primary mouse bone marrow-derived macrophages (BMDM) or mouse embryonic fibroblasts (MEF) infected with *Toxoplasma*, host LD were also observed surrounding the PV (S1 Fig). In HeLa cells overexpressing GFP-ADRP, PV were extensively enveloped with host LD harboring GFP-ADRP at their surface, as compared to uninfected cells in which GFP-ARDP-containing LD were randomly distributed in the cytosol (Fig 2B, panel I). Under conditions of simultaneous OA addition and ADRP overexpression, LD became enlarged, which was more dramatic in infected cells with most of these oversized LD surrounding the PV (Fig 2B, panel II). These data suggest that the attraction of host LD to the PV is a general phenotype of *Toxoplasma* infection *in vitro*.

To scrutinize the host LD-PV interaction in finer detail, we conducted ultrastructural analyses of infected cells incubated with 0.2 mM OA (Fig 2C). By EM, LD are recognizable as relatively homogenous electron-dense structures, without a membranous bilayer. We observed a substantial clustering of host LD at the PV, with LD often organized in multiple layers around the vacuole. The amassing of host LD around the PV and their closeness to the PV membrane did not encumber the physical association of host ER and mitochondria with the PV membrane [39, 40]. The size of host LD in OA-loaded cells was measured, revealing a mean diameter of 0.66 ± 0.15 μm and 1.04 ± 0.56 μm of LD in uninfected and infected cells, respectively (n = 250 LD from 15 cells per condition; *p* < 0.005). Thus, these data indicate an increase in size of the mammalian LD population upon *Toxoplasma* infection, albeit with a greater variation.

We next examined the dynamics of host LD relocation at the PV over time (from 2 h to 32 h p.i.) in ADRP-overexpressing HeLa cells. We considered a PV as positive for host LD association if it had greater than 70% of its perimeter ringed by host LD (Fig 2D). Throughout infection, an accumulation of host LD around the PV was observed. Early in infection (2 h), only ~2% of PV were positive, however, by 8 h p.i., thethe PV percent reached approximately 20. Between 8 h and 24 h, the percent of positive PV increased by 10% yet the PV quadrupled in size, suggesting that the observed increase in the perivacuolar distribution of LD is likely not due to an increase in PV size. The addition of 0.2 mM OA amplified this phenomenon as by 24 h p.i., ~50% of PV were positive. Jointly, these observations highlight the progressive displacement of host LD from a seemingly random location in the cytosol, to the vacuole periphery.

### Host LD and lipolytic activities contribute to *Toxoplasma* development in mammalian cells

**Fig 3:**
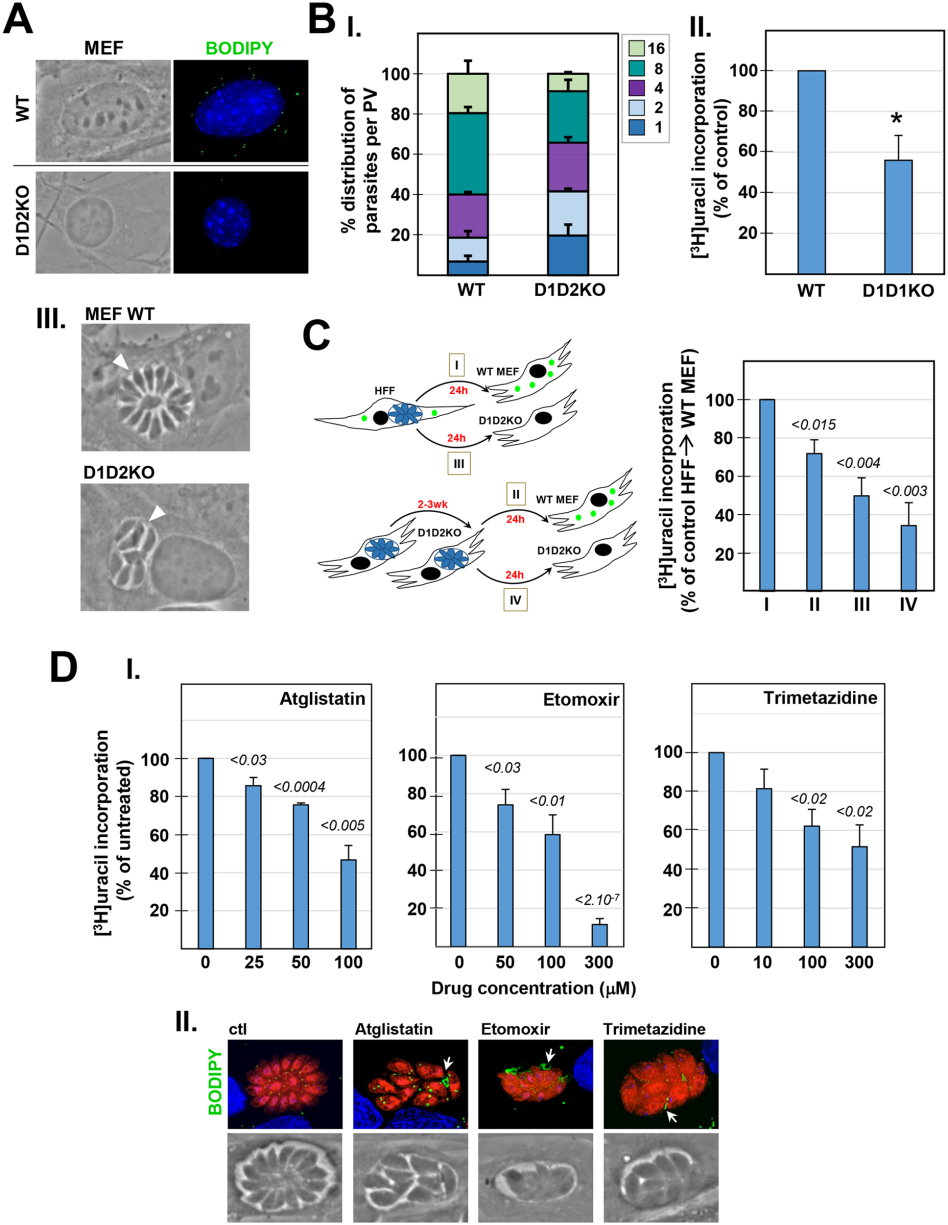
Influence of host LD on *Toxoplasma* intracellular development. **A**. Fluorescence microscopy of uninfected MEF, either WT or lacking *DGAT1* and *DGAT*2 (D1D2KO) stained with BODIPY493/503 (green) and DAPI (blue, nucleus), showing very few, tiny LD in the mutant D1D2KO MEF. **B.** Assessment of parasite development in WT and D1D2KO MEF. The number of parasites per PV in WT and D1D2KO MEF infected for 24 h were counted (panel I). Data are means ± SD of 3 independent experiments. PV sizes were statistically different between the WT and D1D2KO conditions (Chi-squared, *p* <0.0001, all 3 biological replicates significant). Panel II: [^3^H]uracil incorporation assays to quantify parasite replication 24 h p.i. Data are percentages ± SD relative to MEF WT controls (set as 100%) of 4 independent assays performed in triplicate assays (*p <0.0008, Student’s *t* test). Absolute values in cpm for each experiment are shown in S2 Fig. Panel III shows phase images of representative PV in WT and D1D2KO MEF, showing no difference in parasite morphology between the 2 conditions. **C.** Assessment of parasite development following serial passage in D1D2KO MEF. Four experimental conditions were designed as depicted on the schema: *Toxoplasma* from HFF were transferred to WT MET (condition I) or D1D2KO (condition III) for 24 h before [^3^H]uracil incorporation assays. *Toxoplasma* passaged in D1D2KO (P8, P9, P10, or P12) were transferred to WT MEF (condition II) or D1D2KO (condition IV) for 24 h before uracil incorporation assays. Each passage corresponds to a separate biological assay. Data are percentages ± SEM relative to condition I (set as 100%) of 4 biological experiments performed in triplicate assays. Absolute values in cpm (means **±** SD) are shown in S2 Fig. Significant differences in replication rates between condition I and other conditions were calculated using the Student’s *t* test. **D**. Effect of host ATGL, CPT-1 and 3-KAT inhibition on *Toxoplasma* growth. Panel I: *Toxoplasma*-infected HFF were incubated 24 h in the presence of the indicated concentrations of Atglistatin or Etomoxir (both DMSO control), or Trimetazidine (PBS control) before [^3^H]uracil incorporation assays. Data are percentages ± SEM relative to controls (set as 100%) of 3 or 4 biological experiments in triplicates. Significant differences in replication rates between the treated and control samples were calculated with the Student’s *t* test. Absolute values in cpm (means **±** SD) are shown in S2 Fig. Panel II: fluorescence microscopy after 24 h of RFP-*Toxoplasma-*infected HFF stained with BODIPY 493/503 (green) and DAPI (blue), showing representative PV following exposure to Atglistatin (50 μM), Etomoxir (100 μM) or Trimetazidine (100 μM). Smaller PV and lipid deposits in the vacuolar space were observed, compared to untreated parasites.

The redistribution of host LD to the PV and the increased expression of *HsDGAT2* at the onset of parasite replication suggest that host LD may play a role in supporting the intracellular development of *Toxoplasma*. To examine the impact of host LD on *Toxoplasma* infectivity *in vitro*, we infected mutant MEF lacking both *DGAT1* and *DGAT2* [41], which were obtained from a DGAT1 and DGAT2 KO mouse [42, 43]. These mutant cells contain few, tiny LD, as shown by BODIPY 493/503 staining, compared to parental (WT) MEF (Fig 3A). *Toxoplasma* replication rates were quantified by parasite enumeration at 24 h p.i. in DGAT-deficient MEF, and a significant growth delay was observed (Fig 3B, panel I). The slower replication rate of parasites in DGAT-deficient MEF (D1D2KO) was further confirmed by tritiated uracil incorporation into the parasites, showing a growth reduction of ~ 40%, as compared to parasites replicating in WT MEF (Fig 3B, panel II). However, no gross alteration in the morphology of parasites grown in the mutant cells was apparent (Fig 3B, panel III). We next wanted to assess growth defects of *T*. *gondii* across multiple lytic cycles by plaque assays comparing the parasite’s development in D1D2KO MEF and WT MEF. However, this assay was technically impossible due the rapid growth of WT MEF, which filled in the lysis area created by the parasites, resulting in no detectable holes in the cell monolayer. Nevertheless, we were able to observe tiny, circular plaques at day 7 in the monolayer of D1D2KO MEF as these cells grow much slower than WT MEF, suggesting that *Toxoplasma* were able to some extent to complete several lytic cycles in the mutant MEF. To provide quantification of this process, we then explored the impact on *Toxoplasma* replication of repeated cultivation of the parasites, by serial passage, in D1D2KO MEF. We transferred parasites cultivated in D1D2KO MEF for 2 to 3 weeks to either WT or mutant MEF, and measured parasite replication after 24 h by uracil incorporation assays. Controls include parasites cultivated in HFF prior to transfer to either WT or D1D2KO MEF. Confirming our previous findings (Fig 3B, panel II), we observed a ~40–45% growth reduction of parasites in D1D2KO MEF following cultivation in HFF (Fig 3C, conditions I and III). Parasites that have been cultivated for 8 to12 passages in DGAT-deficient MEF then transferred to WT MEF were able to partially recover as their replication rate corresponded to ~75% of that of parasites cultivated in HFF prior to infection in WT MEF (Fig 3C, condition II). By contrast, parasites maintained solely in DGAT-deficient MEF exhibited a growth reduction of ~60% (Fig 3C, condition IV), and this replication defect was more pronounced than that of parasites cultivated only 24 h in DGAT-deficient MEF. This suggests that the presence of host LD influences parasite development as *Toxoplasma* maintained in DGAT-deficient MEF over several lytic cycles displays slower replication rate.

In addition to their lipid cargo, *T*. *gondii* may take advantage of host LD for their lipid-metabolizing enzymes and energy substrates. Lipolysis is the biochemical pathway responsible for the catabolism of TAG, stored in LD, into glycerol and non-esterified fatty acids by lipases. The free fatty acids can subsequently be used as precursors for major lipid synthesis and membrane biogenesis. In LD, ATGL catalyzes the initial step in TAG hydrolysis, generating DAG and a fatty acid. No *ATGL* homolog is present in the *Toxoplasma* genome. To examine whether *Toxoplasma* exploits some lipolytic activities associated with host LD, we incubated infected cells with Atglistatin, a specific inhibitor of ATGL [44]. In cultured mammalian cells, up to 100 μM of Atglistatin inhibits lipolysis by reducing fatty acid mobilization. Incubation of infected cells with Atglistatin at different concentrations for 24 h resulted in a significant decrease in parasite replication, proportional to the drug concentration, as assessed by radioactive uracil incorporation (Fig 3D, panel I).

As *Toxoplasma* attracts both host LD and mitochondria to its PV, we next investigated if the parasite takes advantage of the liberation of fatty acids from the host LD and subsequent oxidation of these fatty acids in the host mitochondria, resulting in the generation of energy substrates and ATP. A central step in the fatty acid β-oxidation cycle is mediated by carnitine palmitoyltransferase-1 (CPT-1), an integral transmembrane protein of the mitochondrial outer membrane that catalyzes the transfer of acyl moieties from CoA to carnitine prior to fatty acyl carnitine internalization into mitochondria [45]. Within the mitochondrial matrix, 3-ketoacyl coenzyme A thiolase (3-KAT) is a key enzyme in the final step of fatty acid ß-oxidation generating acetyl-CoA [46]. *T*. *gondii* lacks both CPT-1 and 3-KAT genes. Etomoxir is an irreversible inhibitor of CPT-1 [47] while Trimetazidine is a competitive inhibitor of 3-KAT [48]. Incubation of *Toxoplasma*-infected cells with Etomoxir or Trimetazidine at various concentrations results in decreased parasite replication, proportional to drug concentrations (Fig 3D, panel I). Microscopy observations of infected cells exposed for 24 h to 50 μM Atglistatin (~20% growth reduction of *Toxoplasma*), 100 μM Etomoxir or 100 μM Trimetazidine (both causing ~40% decreased growth), reveal disorganized parasites within the PV, as compared to untreated conditions (Fig 3D, panel II). BODIPY 493/503 staining illustrates the presence of large lipidic material in the PV lumen, accumulated between the parasites, suggesting lipid metabolic disorders.

The sensitivity of *Toxoplasma* towards the inhibition of host ATGL, CPT-1 and 3-KAT activity suggests that the parasite may take advantage of the lipolytic activity of the host LD and products generated in the fatty acid β-oxidation cascade in host mitochondria.

### *Toxoplasma* accesses and incorporates host lipid droplet-derived lipids

**Fig 4:**
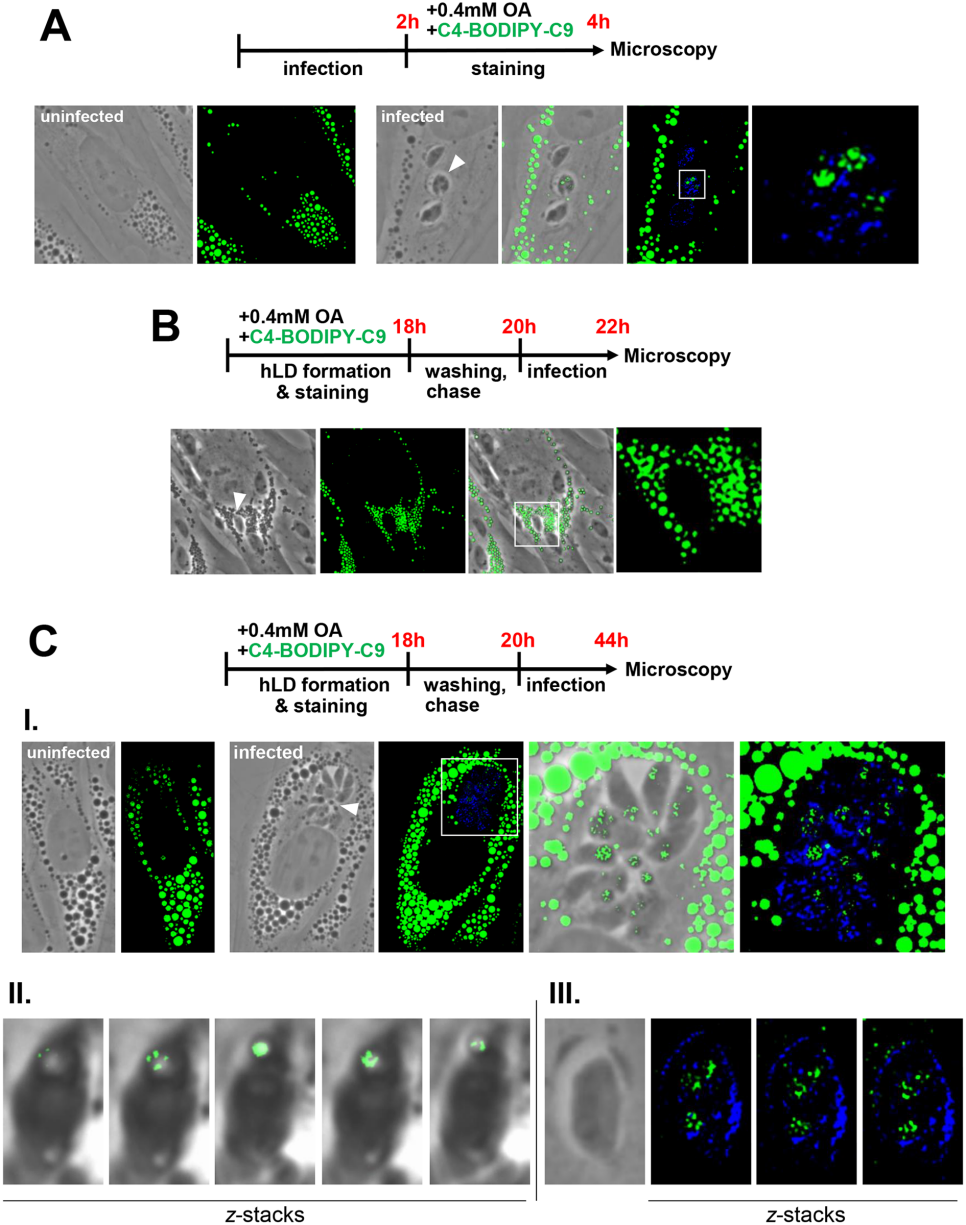
Detection in *Toxoplasma* of the fatty acid C4-BODIPY-C9 present from host LD. **A-C.** Fluorescence microscopy of uninfected or infected HFF in the presence of 0.4 mM OA and the free fatty acid C4-BODIPY-C9 (10 μM). Schemas outlining the experimental protocols are shown at the top of each panel. In A, C4-BODIPY-C9 (green) was co-administrated with OA for 2 h before fixation and immunolabeling of the PV with anti-GRA7 antibodies (blue), resulting in C4-BODIPY-C9 straining on the parasite. In B and C, C4-BODIPY-C9 (green) was co-administrated with OA for 18 h prior to infection, allowing the storage of C4-BODIPY-C9 in host LD (hLD). After washing and C4-BODIPY-C9 chase, cells were infected for 2 h (B) or 24 h (C). C4-BODIPY-C9 association with the parasite was apparent at 24 h (C, panel I). Panel II in C shows *z*-stacks of egressing parasite containing C4-BODIPY-C9 on LD as observed by live fluorescence microscopy. Panel III in C shows *z*-stacks of intracellular parasite stained for GRA7 (blue) and containing C4-BODIPY-C9 on cytoplasmic membranes by IFA.

The redistribution and closeness of host LD to the PV membrane suggests that *Toxoplasma* may take advantage of mammalian LD for their lipid stores and/or metabolic properties. We performed assays to examine whether *Toxoplasma* is able to take up neutral lipids from host LD. In lipid trafficking studies, BODIPY-fatty acids are routinely used and have been shown to incorporate into LD and be readily metabolized by living cells to phospholipids, DAG, TAG and CE [49–52]. We incubated infected cells with C4-BODIPY-C9, which has the fluorophore BODIPY 500/510 linked within the fatty acid chain, to track the internalization of this fatty acid analog into the parasite. A previous study on *Toxoplasma*-infected cells incubated with C4-BODIPY-C9 showed fluorescence staining associated with the parasite [53]. In our experimental system in which infected cells were incubated with C4-BODIPY-C9 premixed with 0.4 mM OA, a condition leading to the overaccumulation of fluorescent LD, we also detected fluorescent staining inside the parasite (Fig 4A). To specifically assess whether the parasite retrieves fluorescent lipids from host LD, we pre-incubated HFF with the C4-BODIPY-C9/0.4 mM OA mixture for 18 h, washed away C4-BODIPY-C9 and infected with *Toxoplasma* for 2 h or 24 h. After 2 h of infection, no fluorescent signal was apparent within the PV or in the parasite, based on live cell microscopy (Fig 4B). However, after 24 h of infection, we observed a fluorescent staining within the parasite, illustrating that *Toxoplasma* accumulated C4-BODIPY-C9 or lipids derived from it (Fig 4C, panel I). Some fluorescent foci appeared reminiscent of parasite LD [22, 23], further evidenced on successive fluorescent *z*-slice images of an egressing parasite (Fig 4C, panel II). The fluorescence staining was also observed on intracellular membranes, suggesting that the parasite can readily incorporate LD-derived lipids as building blocks into organellar membranes (Fig 4C, panel III). Together, these results suggests the trafficking of C4-BODIPY-C9 from host LD to the parasite interior by 24 h p.i. Nevertheless, the possibility that the parasite internalizes C4-BODIPY-C9-derived lipids from other sources in the host cell besides LD, cannot be excluded.

**Fig 5:**
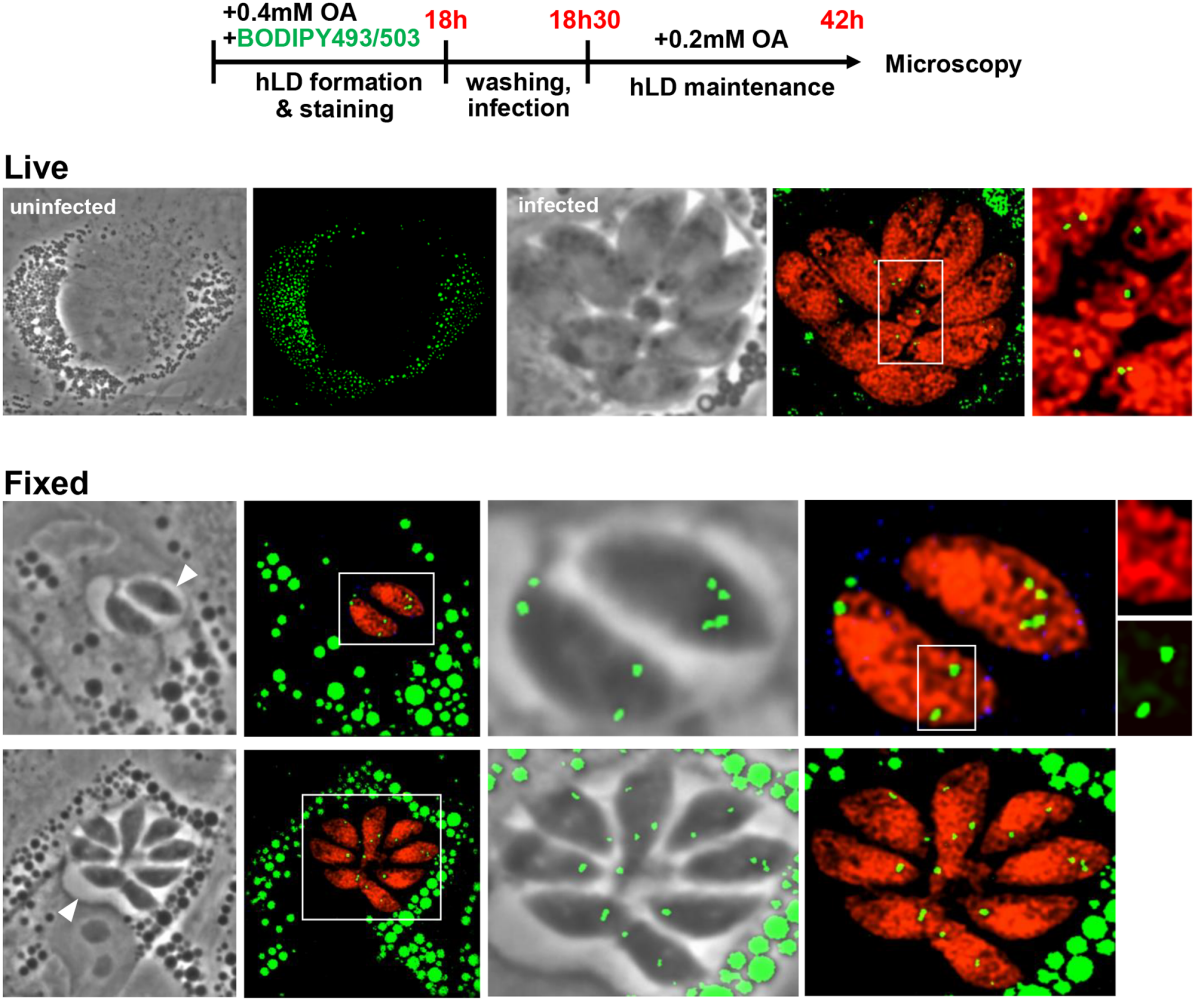
Detection in *Toxoplasma* of BODIPY 493/503 originated from host LD. Fluorescence microscopy of uninfected or infected HFF with RFP-expressing parasites in the presence of 0.4 mM OA and BODIPY 493/503 (10 μM). Schema outlining the experimental protocols is shown at the top. BODIPY 493/503 (green) was co-administrated with OA for 18 h prior to infection, allowing the storage of BODIPY 493/503 in host LD (hLD). After washing and infection in the presence of 0.2 mM OA, to maintain host LD, for ~24 h, cells were observed by live microscopy (upper panel) or fixed for IFA using anti-GRA7 antibodies (in blue; lower panel). In both cases, the BODIPY 493/503 signal was observed within the parasite both in small and large PV (arrowheads).

Therefore, to ensure that the parasite accessed and incorporated, into its PV and cytoplasm, neutral lipids originating from host LD, we devised an experimental protocol in which the non-metabolizable lipid dye BODIPY 493/503 was stored in host LD prior to and during infection. Host LD formation in HFF was stimulated by adding 0.4 mM OA mixed with BODIPY 493/503 for 18 h, which resulted in a plethora of fluorescent host LD (Fig 5). After washing, HFF pre-loaded with BODIPY-stained LD were infected with RFP-expressing *Toxoplasma* for 24 h in the presence of 0.2 mM OA, to pressure the host cell to maintain LD, and viewed by live or fixed microscopy. Remarkably, BODIPY 493/503 staining was observed within the parasite, mostly accumulated within their LD, and the fluorescent signal was more prominent in fixed parasites. As this staining could only have occurred if the parasite internalized fluorescent lipids derived directly from host LD prior to storage in their own LD, this data establishes that *Toxoplasma* is able to access lipids confined to host LD.

### *Toxoplasma* sequesters host Rab7-associated lipid droplets into the PV

**Fig 6:**
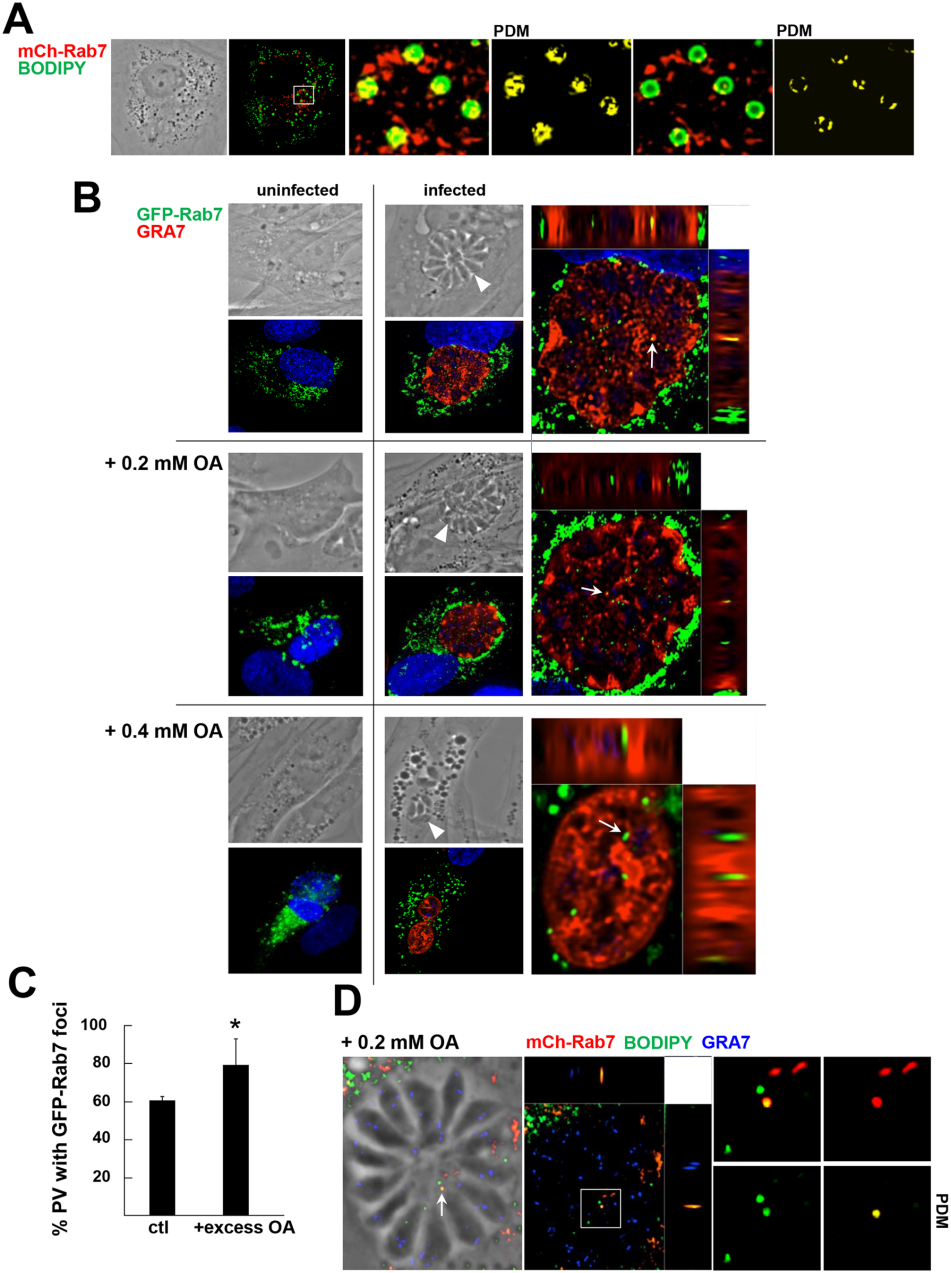
Detection of host Rab7-associated structures in the PV. **A.** Fluorescence microscopy of HFF expressing mCherry-Rab7. mCherry Rab7-expressing HFF incubated with 0.2 mM OA for 24 h were fixed and stained with BODIPY 493/503. A phase and a maximum projection (extended depth of field) image displaying the merge of BODIPY 493/503 (green) and mCherry Rab7 (red) are shown. A digital magnification of the boxed area is shown as two individual *z*-slice images displaying the merge of BODIPY 493/503(green) and mCherry Rab7 (red) plus the positive PDM, showing a subset of host LD containing Rab7 on their surface. **B.** Fluorescence microscopy of uninfected or 24 h-*Toxoplasma*-infected HFF expressing GFP-Rab7 grown without OA, with 0.2 or 0.4 mM OA. Cells were fixed and stained with antibodies for GRA7 (red; PV) and DAPI (blue; nucleus). Arrowheads pinpoint PV on phase images. The distribution of GFP-Rab7-positive vesicles (green) is shown in both uninfected and infected cells. A digital magnification of the *Toxoplasma* PV is shown in an orthogonal view of the z-stack to highlight the localization of host-derived GFP-Rab7 vesicles inside the PV (arrows). **C.** Quantification of the percentage of PV containing GFP-Rab7-associated structures in the PV lumen. Infected HFF expressing GFP-Rab7 were incubated in the absence of added OA (control) or with excess OA (0.2 or 0.4 mM) as described in B. The number of PV containing GFP-Rab7 structures was determined by analysis of microscopy images of optical *z*-stacks. Data are mean values ± SD (n = 3) (PV > 20 in each experiment). Values are statistically significant between PV exposed to OA (0.2 or 0.4 mM) and without OA as control (Chi-squared test, *p <0.0129). **D.** Fluorescence microscopy of mCherry-Rab7 expressing HFF infected with *Toxoplasma* for 24 h, and stained with BODIPY 493/503 (green) and GRA7 (blue). A phase and orthogonal view of a fluorescent *z*-stack are shown. A digital magnification of the boxed region displayed as individual *z*-slices showing the BODIPY 493/503 channel (green), the mCherry-Rab7 channel (red), a merged image and positive PDM. These images illustrate the co-staining of intravacuolar structures for mCherry-Rab7 and BODIPY 493/503 (arrow).

Next, we focused on the cellular mechanism involved in the delivery of neutral lipids stored in host LD to the PV and the parasite. We previously demonstrated that *T*. *gondii* diverts and sequesters into the PV host mammalian Rab14, Rab30 and Rab43 vesicles containing lipids [24]. In mammalian cells, several Rab GTPase proteins are detected on the LD surface, such as Rab7 involved in lipophagy [54] and Rab18 [55]. We investigated whether Rab7- or Rab18-associated LD were selectively re-routed to the PV allowing *Toxoplasma* access to their cargo of neutral lipids. We first confirmed that Rab7 localizes, at least in part, to LD by transfecting HFF with a plasmid containing mCherry-Rab7 and staining with BODIPY 493/503. Partial colocalization (PCC: 0.129) of mCherry-Rab7 and BODIPY 493/503 was observed, primarily on the surface of the LD (yellow, positive difference from the mean (PDM) image) (Fig 6A). We also detected mCherry-Rab7 and BODIPY 493/503 signals partially co-localized in *Toxoplasma*-infected HFF (S3 Fig). Upon the addition of OA, the number of foci containing the two fluorescent signals significantly increased; measurement of the fluorescence overlap was performed by counting the number of BODIPY 493/503 foci with or without mCherry-Rab7 in the presence or the absence of 0.2 mM OA, and a ~3-fold increase in yellow foci was calculated (*p* < 0.005 in uninfected vs. infected cells).

We next devised assays to search for the presence of Rab7 foci within the PV of *Toxoplasma*. GFP-Rab7-expressing HFF were infected with *Toxoplasma* for 24 h with or without addition of OA and immunostained for GRA7, to delineate the PV membrane (Fig 6B). In all conditions, several GFP-Rab7 foci were observed inside the PV, as visualized in the orthogonal view of the *z*-stack. The percent of PV with intravacuolar GFP-Rab7 foci significantly increased in the presence of 0.2 or 0.4 mM OA added to the medium (Fig 6C). However, to rule out the possibility that these foci are Rab7-associated endocytic vesicles rather than LD, we repeated the assay on infected mCherry-Rab7-expressing HFF, stained with BODIPY 493/503 (Fig 6D). Intravacuolar foci stained for both mCherry-Rab7 and BODIPY 493/503 were observed in 3D reconstructed images, shown both as an orthogonal view of a *z*-stack and as individual *z*-slice images. This indicates that these yellow foci, which correspond to *bona fide* host LD, were sequestered into the PV. In infected GFP-Rab18-expressing HFF, we also observed intravacuolar GFP-Rab18-positive structures (S4A Fig) inside ~40% of *Toxoplasma* PV (S4B Fig). In contrast to GFP-Rab7, there was no significant increase in the percent of PV containing Rab18 in the presence of excess OA.

The observations suggest that *Toxoplasma* may take up LD via the recognition of Rab7, and potentially Rab18, which may be a mechanism by which the parasite accesses neutral lipids stored in host LD.

### Host LD protrude into the PV of *Toxoplasma* and penetrate the PV lumen

**Fig 7:**
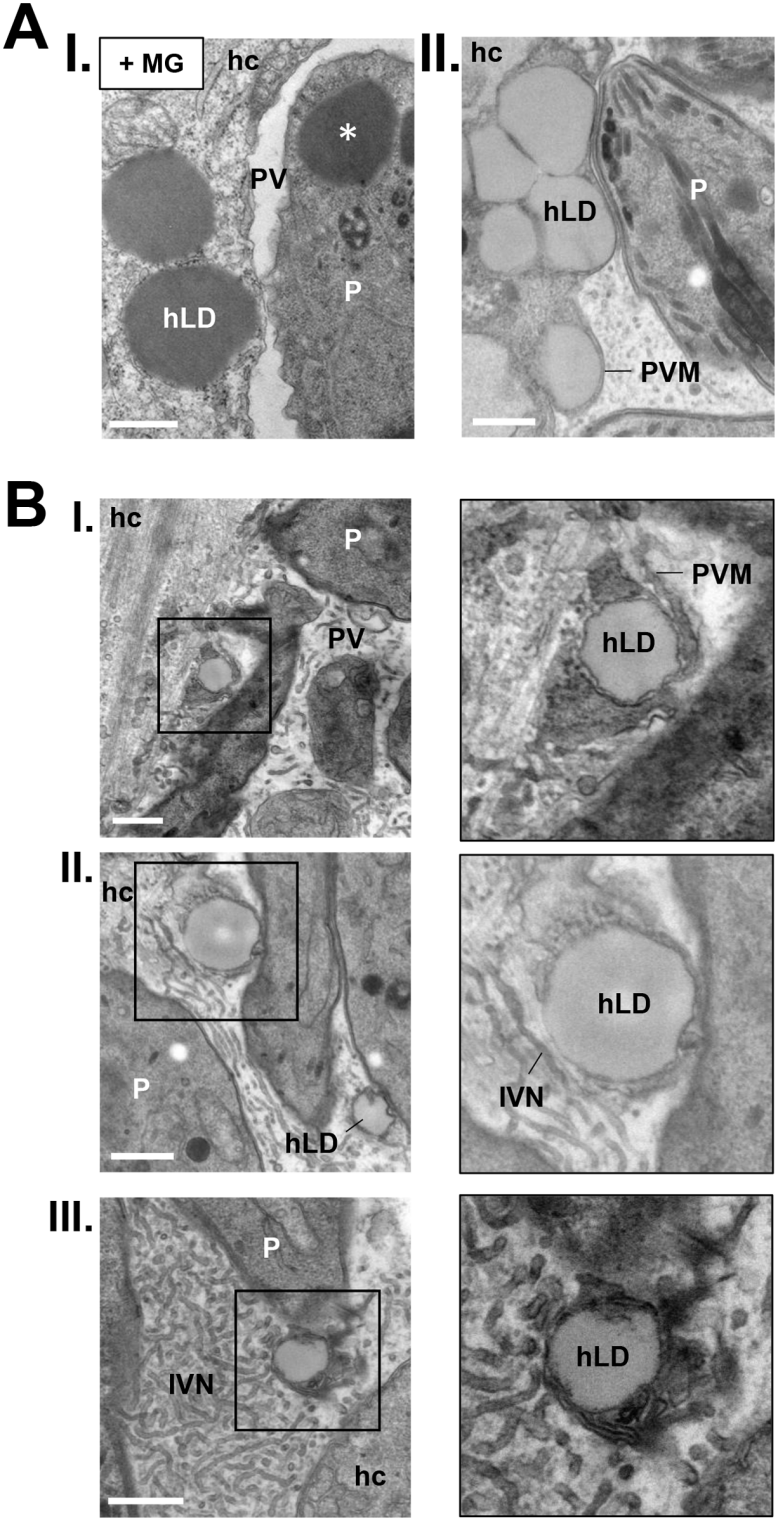
Ultrastructural evidence of host LD projection and trapping into the PV. **A**-**B.** Transmission EM of *Toxoplasma*-infected HFF for 24 h. In A, panel I: infected cells were fixed in the presence of malachite green (MG) to facilitate the identification of LD while in panel II, infected cells were incubated with 0.2 mM OA to increase LD number. In both situations, host LD (hLD) were observed protruding from the host cytoplasm into the PV lumen. The asterisk points to parasite LD. In B, infected cells were loaded with 0.2 mM OA and host LD were observed in the vacuolar space, wrapped by IVN tubules (panels I-III). hc, host cell; P, parasite; PVM, PV membrane. All scale bars, 0.5 μm.

The above observations prompted us to conduct ultrastructural studies to ascertain the presence host LD inside the PV of *Toxoplasma*, and to provide detailed information on their ultrastructure and specific location inside the vacuole. HFF were infected with *Toxoplasma* for 24 h and then fixed in the presence of malachite green, an organic compound which retains lipid elements [56], resulting in the preservation and enhanced staining of lipid-rich structures such as LD (Fig 7A, panel I). In many instances, we observed several host LD not only in close proximity to the PV membrane, but also protruding into the PV. In HFF infected with *Toxoplasma* for 24 h incubated with 0.2 mM OA, even more host LD were observed bulging against the vacuolar membrane (Fig 7A, panel II). Examination of the PV content reveals the presence of LD inside the vacuole (Fig 7B), some of them surrounded by the PV membrane (Fig 7B, panel I). These LD displayed all the features of LD found in the host cytoplasm, including a thin phospholipid monolayer, and a similar size, electron-density and shape (S5 Fig), confirming that host LD were translocated from the host cytosol to the intravacuolar space.

The PV lumen contains thin tubules organized in a dense network named the intravacuolar network (IVN). The IVN is initially secreted by the parasite [57] and then expanded through the salvage of host lipids by the parasite via an unknown mechanism [58]. It has been proposed that this network may be involved in nutrient uptake [59], or parasite protein targeting to the PV membrane [60]. Interestingly, host LD internalized into the PV were wrapped by tubules of the IVN, as illustrated for 3 LD (Fig 7B, panels II and III).

### The IVN contributes to lipids delivery from host LD to the parasite

**Fig 8:**
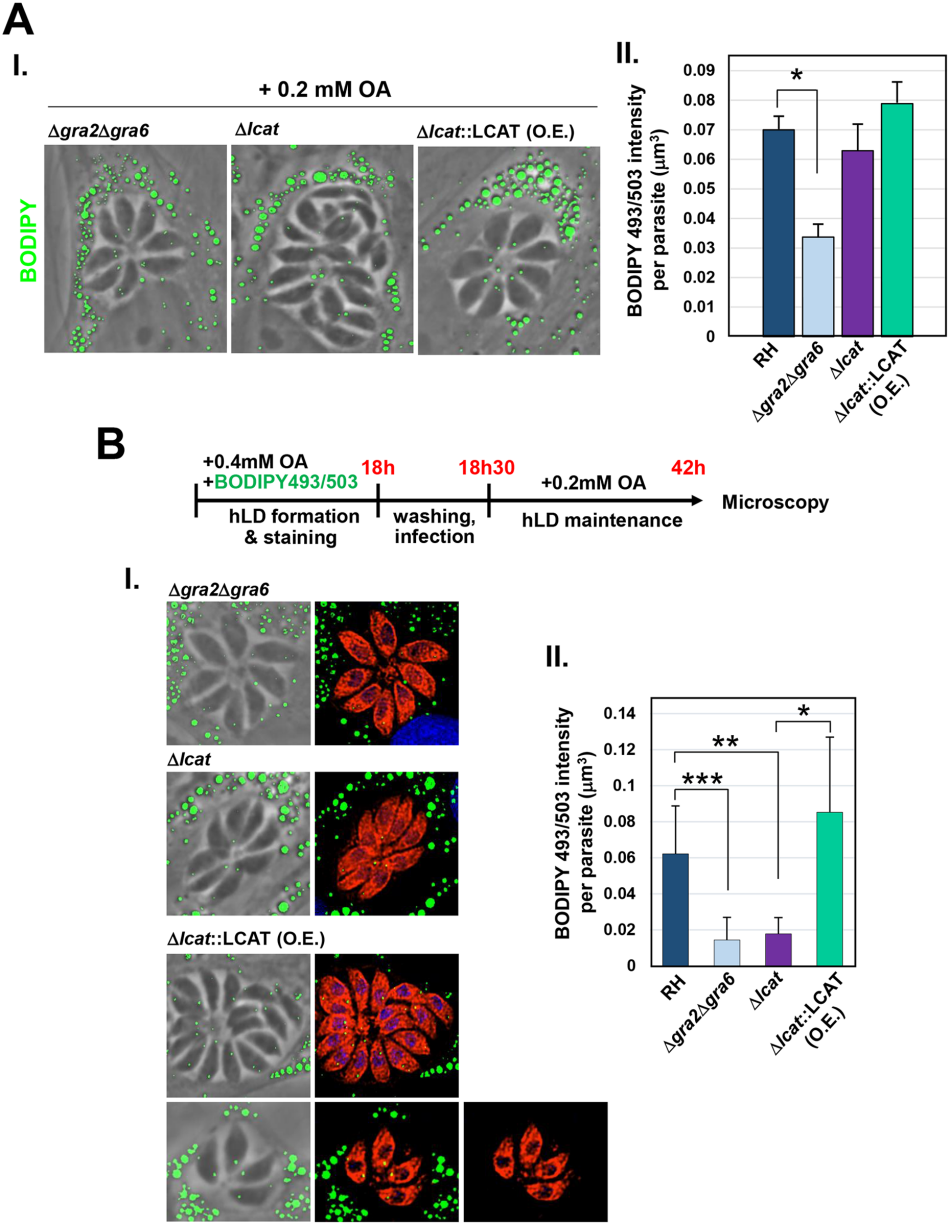
Detection of BODIPY 493/503 originated from host LD in *Toxoplasma* mutants. **A.** Fluorescence microscopy of HFF infected with Δ*gra2*Δ*gra6*, Δ*lcat* and Δ*lcat*::LCAT overexpressors (O.E.) for 24 h with 0.2 mM OA added to the medium. Host LD were identified by staining with BODIPY 493/503 (green) overlaid on phase contrast as shown in panel I. Panel II: the basal LD volume of the parasites was calculated by measuring the intensity of BODIPY 493/503 within individual PV, and normalized to parasite number, without the addition of OA (means ± SD of triplicate experiments, n > 400 parasites per experimental replicate and mutant (*t*-test: *p <0.01). **B.** Fluorescence microscopy of infected HFF with Δ*gra2*Δ*gra6*, Δ*lcat* and Δ*lcat*::LCAT O.E. parasites in the presence of 0.4 mM OA and BODIPY 493/503 (10 μM). Schema outlining the experimental protocols is shown at the top. BODIPY 493/503 (green) was co-administrated with OA for 18 h prior to infection, allowing the storage of BODIPY 493/503 in host LD (hLD). After washing and infection in the presence of 0.2 mM OA, to maintain host LD for 24 h, cells were fixed for IFA using antibodies against anti-Hsp70/aldolase antibodies to stain the parasite cytosol (in red). Panel I shows representative PV for Δ*gra2*Δ*gra6* and *Δlcat* parasites for which the BODIPY 493/503 signal was weak as compared to Δ*lcat*::LCAT overexpressors (large and small PV). Panel II: the average LD volume of the parasites was calculated using Volocity to determine the averaged BODIPY 493/503 intensity, thus volume, within parasites. Data are means of means of 3 independent experiments, n > 400 parasites per experimental replicate and mutant (*t*-test: ***p <0.008; **p <0.02; *p <0.04).

The wrapping of host LD within the membranous structures of the IVN urged us to investigate whether this network is important for lipid availability to the parasite through the sequestration of host LD in the PV. To test this hypothesis, we exploited a mutant parasite lacking two main structural proteins located to the IVN, GRA2 and GRA6, which are both essential for the formation and stabilization of the IVN [61]. We first examined the intracellular distribution of host LD in fibroblasts infected with the double knockout Δ*gra2*Δ*gra6* in the presence of 0.2 mM OA. Host LD recruitment around the PV of the mutant was observed (Fig 8A, panel I), similarly as for WT parasites (Fig 2A, panel II). Next, we looked at the capability of the parasite mutant to store lipids and form LD by staining infected cells with BODIPY 493/503. Quantification of the volume of the green fluorescent signal within individual parasites reveal significantly less staining (~50% reduction) in the Δ*gra2*Δ*gra6* mutant, as compared to WT parasites (Fig 8A, panel II). The ability of the Δ*gra2*Δ*gra6* strain to retrieve lipids directly from host LD was then probed using the protocol as described in Fig 5. Mutant parasites were capable of scavenging host LD-derived neutral lipids, albeit to a much lower extent than WT parasites, as evidenced by microscopic observations illustrating very spare and small LD (Fig 8B, panel I). Quantification of the fluorescent signal within the mutant reveals BODIPY 493/503 intensity ~ 4-times lower than calculated for WT parasites (Fig 8B, panel II).

We previously characterized the properties of a phospholipase A_2_ enzyme (TgLCAT), a dense granule protein that is secreted by *Toxoplasma* into the PV and is associated with the IVN post-secretion [59]. One attractive hypothesis is that the IVN may serve as a platform for degradative or lipolytic activities mediated by TgLCAT, thus for the processing of host nutrient-enriched structures sequestered into the PV, to cover the parasite’s nutritional needs (manuscript in preparation, JDR, SJN, KE, CP, EH, RH and IC). We previously generated a parasite mutant lacking TgLCAT (Δ*lcat*), which exhibited growth defects both *in vitro* and in mice, and a parasite complemented strain overexpressing TgLCAT (Δ*lcat*::LCAT), which was more virulent in mice than WT parasites [59]. In fibroblasts infected with either Δ*lcat* parasites or TgLCAT-overexpressing parasites, host LD also clustered around the PV (Fig 8A, panel I). Additionally, BODIPY 493/503 staining of infected HFF show that these two parasites strains possessed LD (Fig 8A, panel II). However, by tracking the incorporation of BODIPY 493/503 preloaded in host LD into these parasite strains, a weak BODIPY fluorescent staining was observed in the Δ*lcat* mutant (Fig 8B, panel I), concomitant with a significant ~4- and 5-fold reduction in intraparasitic BODIPY volume as compared to WT and TgLCAT overexpressor parasites, respectively (Fig 8B, panel II). TgLCAT-overexpressing parasites exhibited large LD which stained with BODIPY 493/503 (Fig 8B, panel I), however, BODIPY volume quantification revealed no significant difference with WT parasites (Fig 8B, panel II).

All together, these results suggest that the IVN and, at least, one lipase associated to this network are implicated in the processing of host LD trapped into the PV, which would result in lipid availability for the parasites.

### In the presence of excess OA in the host cell, *Toxoplasma* accumulates acylglycerols and cholesteryl esters

Exogenously added OA at physiological concentrations up to 0.5 mM leads to a net synthesis of TAG and activation of the TAG/free fatty acid cycling, which protect mammalian cells against lipotoxicity [62]. In our system using fibroblasts, addition of 0.2 mM OA to the medium of a confluent layer of cells for 24 h resulted in an increase in transcription of *hsADRP* by 3.9-fold, *hsDGAT1* by 1.7-fold, and *hsDGAT2* by 1.9-fold, as compared to untreated cells (S6 Fig). No statistically significant increase in either *hsACAT1* or *hsATGL* gene expression was observed.

**Fig 9:**
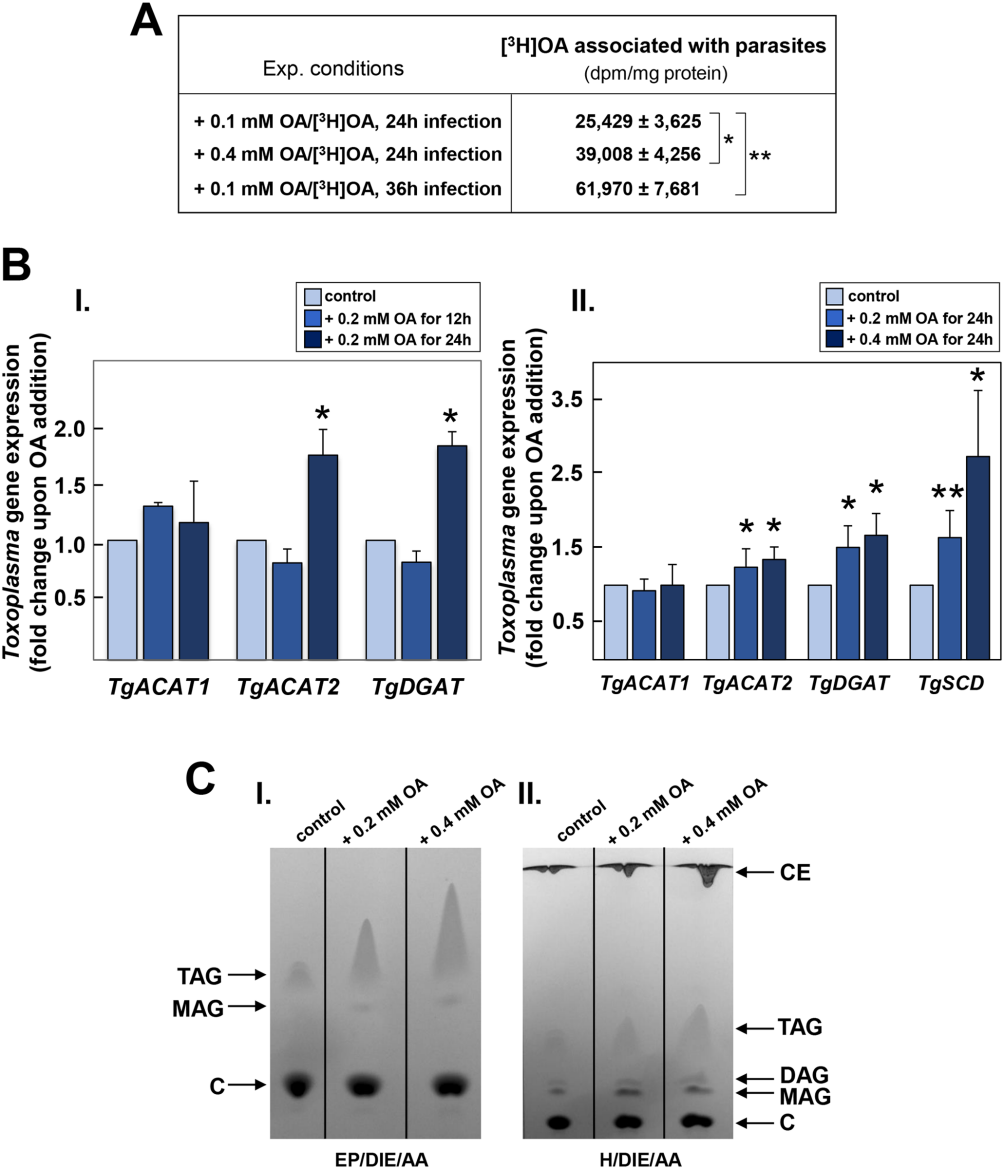
Influence of excess oleate on parasite neutral lipid synthesis. **A.** Uptake assays of radioactive OA by *Toxoplasma*. HFF were preincubated with 0.1 or 0.4 mM OA containing [^3^H]OA for 24 h, thoroughly washed and infected with *Toxoplasma* for 24 h or 36 h before chemically inducing parasite egress. Parasites were collected and purified to monitor their radioactivity by counting. Data of [^3^H]OA associated with the parasites expressed in dpm/mg cell protein, are means ± SD of 3 independent assays made in triplicates. *p <0.05; **p <0.001. **B.** Comparison of *Toxoplasma* ACAT and DGAT gene expression under conditions of excess OA or without added OA (control). Real-time PCR analysis of TgACAT1, TgACAT2, TgDGAT and TgSDC transcripts in parasites infecting cells exposed to 0.2 mM OA for 12 h or 24 h (panel I), or to 0.2 or 0.4 mM for 24 h (panel II). Data are means ± SD of 3 independent assays performed in triplicates. Transcripts levels of *TgACAT2* and *TgDGAT* were significantly increased in the presence of excess OA compared to control conditions in the absence of OA (*p <0.004; **p <0.0001). **C.** TLC analysis of neutral lipid fractions of cellular lipid extracts from intracellular *Toxoplasma* exposed to excess OA. Neutral lipids were separated on plates using as solvent EP/DIE/AA (panel I) to resolve TAG and MAG, or H/DIE/AA (panel II) to separate CE and DAG. CE and species of acylglycerols were more abundantly produced under conditions of excess OA vs. no OA added.

Next, we examined the response of *Toxoplasma* to excess OA added to the medium and/or to excess TAG in host LD. Intracellular *Toxoplasma* is able to divert various fatty acids present in its environment [53, 63, 64]. Upon the exogenous addition of excess OA during infection, the parasite may salvage OA as free fatty acid and/or as OA-containing lipids from many host cell compartments including LD. First, we investigated the extent to which *Toxoplasma* was able to take up OA packed in host LD. LD formation was triggered by incubating HFF in either 0.1 mM or 0.4 mM OA, both mixed with traces of [^3^H]OA, for 24 h. Cells were washed and infected with *Toxoplasma*, and 24 h or 36 h p.i, parasite egress was induced using the calcium ionophore A23817 [65]. Parasites were then collected and purified from cellular debris before assessing their radioactivity by scintillation counting (Fig 9A). Tritiated material was incorporated into *Toxoplasma* proportionally to the OA concentrations and the infection times, revealing the avidity of the parasite to divert large amounts of lipids accumulated in its environment.

Like many organisms, *T*. *gondii* encodes enzymes to form TAG and CE for storage in its own LD [22, 23, 63]. In contrast to mammalian cells, *Toxoplasma* possesses two ACAT enzymes (TgACAT1 and TgACAT2) that are both involved in producing CE for storage in LD. While TgACAT1 preferentially utilizes palmitoyl-CoA, TgACAT2 has broader fatty acid specificity and produces more CE. The parasite expresses only one DGAT, TgDGAT responsible for all its TAG synthesis. Second, we examined whether *T*. *gondii* activates its enzymatic machinery for TAG and CE synthesis in order to counter the uptake of excess OA by measuring the expression levels of *TgACAT1*, *TgACAT2* and *TgDGAT* in intracellular parasites exposed to excess OA for 12 h or 24 h. Minor, statistically insignificant variations were observed in all three transcripts at 12 h p.i. as compared to control conditions without OA addition (Fig 9B, panel I). However, at 24 h p.i., a significantly increase in *TgACAT2* (1.7-fold) and *TgDGAT* (1.8-fold) expression was observed. As *T*. *gondii* salvages exogenous OA proportionally to its extracellular concentration, we extended our transcriptional analysis to intracellular parasites exposed to 0.4 mM OA for 24 h, and unsurprisingly, we detected higher levels of TgACAT2 and TgDGAT transcripts under this condition (Fig 9B, panel II).

Unsaturated fatty acids, such as OA, are synthesized from saturated fatty acids by the enzymatic activity of desaturases [66]; for example, the SCD enzyme (Δ-9 fatty acid desaturase) catalyzes the synthesis of monounsaturated fatty acids including OA, via the introduction of a single double bond [67–69]. In mammalian cells, intracellular levels of fatty acids are controlled, in part, by SCD. The addition of 0.1 mM OA in the medium down-regulates SCD mRNA expression in many cells types, excluding adipocytes in which SCD expression is associated with lipogenesis induction and TAG storage [70–73]. This leads to the question of whether *Toxoplasma* encodes a SCD orthologue and, if so, how its expression is controlled in the presence of excess OA. Genome searches in *T*. *gondii* reveal the presence of a gene coding for a single SCD (Δ-9 fatty acid desaturase; TGME49_238950 in www.toxodb.org), potentially involved in the synthesis of stearate to oleate. To examine whether TgSCD is regulated in the presence of excess OA, we measured the transcription of *TgSCD* in intracellular parasites incubated with excess OA. *TgSCD* transcription was upregulated by 1.65- and 2.7-fold in the presence of 0.2 and 0.4 mM OA respectively, which is analogous to the response of adipocytes exposed to excess OA.

To verify whether the upregulated transcriptional activities of *TgACAT2* and *TgDGAT* in parasites exposed to excess OA correlate with increased activities of ACAT and DGAT enzymes, we measured the amounts of neutral lipids produced by intracellular parasites following exposure to 0.2 and 0.4 mM OA for 24 h. In an initial thin layer chromatography (TLC) analysis of parasite lipid extracts separating the least polar lipids, higher amounts of TAG were detected for *T*. *gondii* incubated with excess OA, with an ~3- and ~4.5-fold increase for 0.2 and 0.4 mM OA, respectively, as compared to control condition (Fig 9C, panel I); no quantitative changes were observed for cholesterol among experimental conditions. In a second TLC migration where a lower-polarity solvent was used to separate apolar components, higher quantities of CE were detected for *T*. *gondii* incubated with excess OA, with an ~1.5- and ~2.5-fold increase with 0.2 and 0.4 mM OA, respectively; slightly higher amounts of monoacylglycerols (MAG) and DAG were also observed with 0.2 or 0.4 mM (Fig 9C, panel II). In conclusion, OA salvaged by *T*. *gondii* is mainly converted to TAG and CE, which is consistent with the upregulation of *TgACAT2* and *TgDGAT* in the presence of excess OA. It remains possible, however, that the large amounts of TAG detected in the parasites may also be salvaged as TAG molecular species from host LD, instead of OA salvaged and converted to TAG within the parasite.

### Upon excess OA, *Toxoplasma* packages large quantities of neutral lipids in lipid droplets

**Fig 10:**
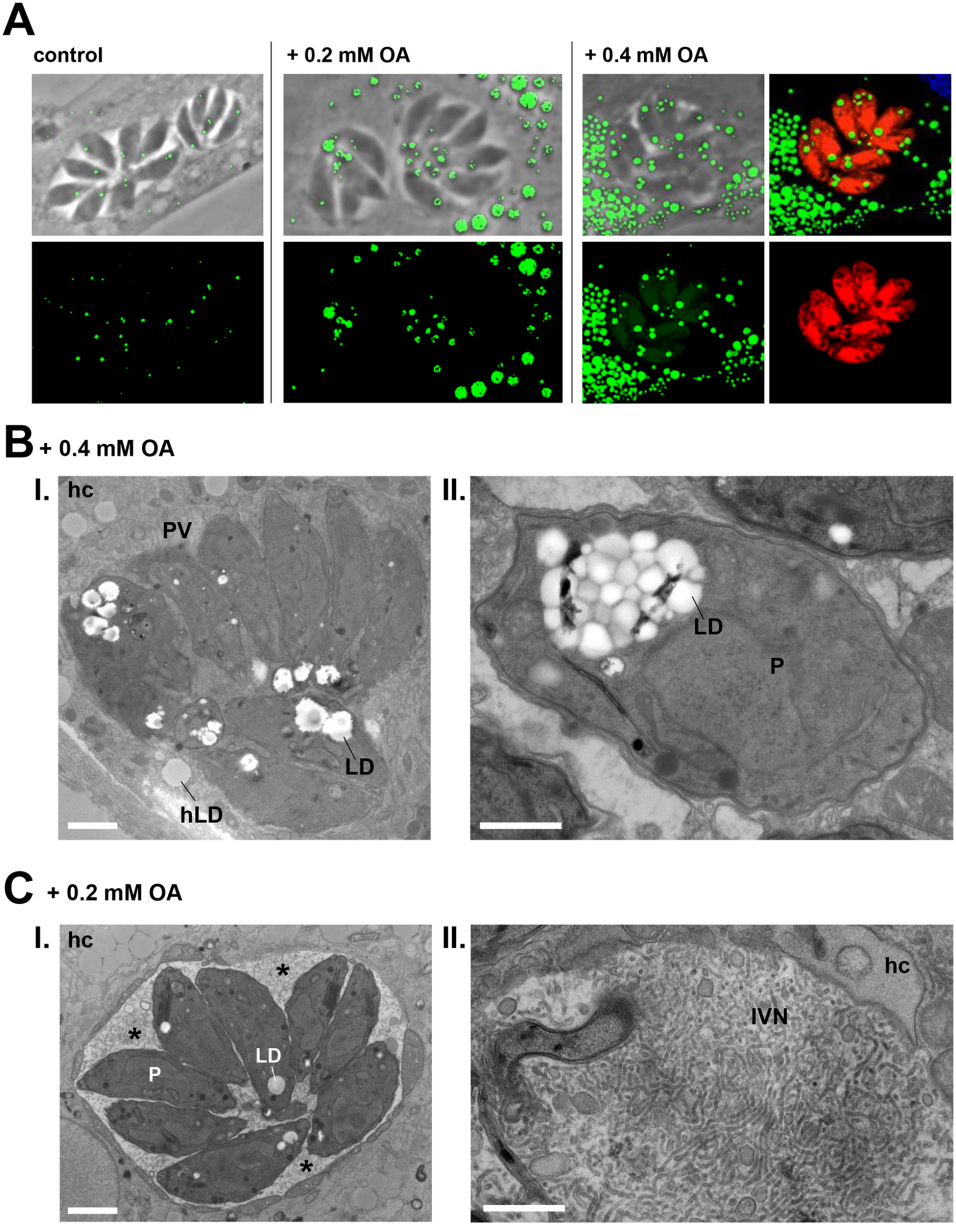
Influence of excess oleate on parasite neutral lipid stores and IVN size. **A.** Fluorescence microscopy of RFP-expressing *Toxoplasma*-infected HFF. Infected cells were cultivated without OA (control), or with 0.2 mM OA for 24 h or 0.4 mM OA for 40 h before staining with BODIPY 493/503 (green). Large, fluorescent neutral lipid deposits inside *Toxoplasma* were observed in the presence of 0.4 mM OA. These deposits are confirmed by the presence of cytoplasmic exclusion zones of the RFP fluorescence in the parasite. **B.** Transmission EM images of HFF infected for 24 h in the presence of 0.4 mM OA, showing many parasite LD with some as large as host LD (hLD; panel I) and others clustered inside the parasite (panel II). Scale bars, 0.5 μm. **C.** Transmission EM images of a *Toxoplasma* PV inside HFF infected for 24 h in the presence of 0.2 mM OA. Panel I (asterisks) shows the expansion of the IVN throughout the vacuolar space, taking up all available space between parasites while panel II shows a magnified view focusing on the abundance of long, intertwined tubules. P, parasite; hc, host cell. Scale bars, 0.3 μm.

As our TLC analyses indicate an accumulation of various neutral lipids into *Toxoplasma* following excess OA incubation and that the majority of neutral lipids in many organisms are predisposed to be stored in LD, we stained infected cells with BODIPY 493/503 to inspect the parasite’s lipid stores. Under normal culture conditions, each parasite contains an average of 2 LD with a mean diameter of ~0.2 μm [22]. However, after incubation with 0.2 and 0.4 mM OA for 24 h, we detected up to 8 fluorescent puncta within the parasite (Fig 10A). At 0.4 mM OA, the fluorescent signals were more intense and the puncta were larger, occupying a large volume of the parasite’s cytosol (Fig 10A); some of these fluorescent puncta were as large as host LD, with a diameter up to ~0.6 μm. These large fluorescent structures may correspond to either several immense LD or a cluster of many smaller LD. To resolve this issue, we conducted EM studies on infected cells incubated with 0.4 mM OA. We observed a gathering of many small LD (up to 20 per parasite) in the cytosol more frequently than isolated gigantic LD (Fig 10B, panels I and II). Thus, in response to excess OA, the parasite is able to stimulate LD biogenesis for the storage of large quantities of neutral lipids.

The PV lumen contains an IVN of tubules that are secreted *en bloc* by the parasite into the PV [57]. A previous study suggests that host lipids are also major contributors to the IVN [58]. To determine whether excess OA affects the IVN, we analyzed by EM the PV content of parasites infecting cells incubated with 0.2 mM OA. In the absence of added OA, the IVN remains confined to the middle of the PV, and sometimes between a few parasites but it does not fill the entire available space within the PV [57]. By contrast, in the presence of 0.2 mM OA, the IVN dramatically expands and occupies the entire vacuolar space available between parasites (Fig 10C, panel I). Higher magnification images of PV in the presence of excess OA illustrate the extremely compacted structure of the IVN that is composed of remarkably long, intertwined tubules (Fig 10C, panel II). These findings suggest that part of the OA salvaged by *Toxoplasma* is incorporated into the IVN, contributing to the dramatic expansion of this network.

### Exogenous addition of 0.2 mM OA reveals novel endocytic-like structures in *Toxoplasma*

**Fig 11:**
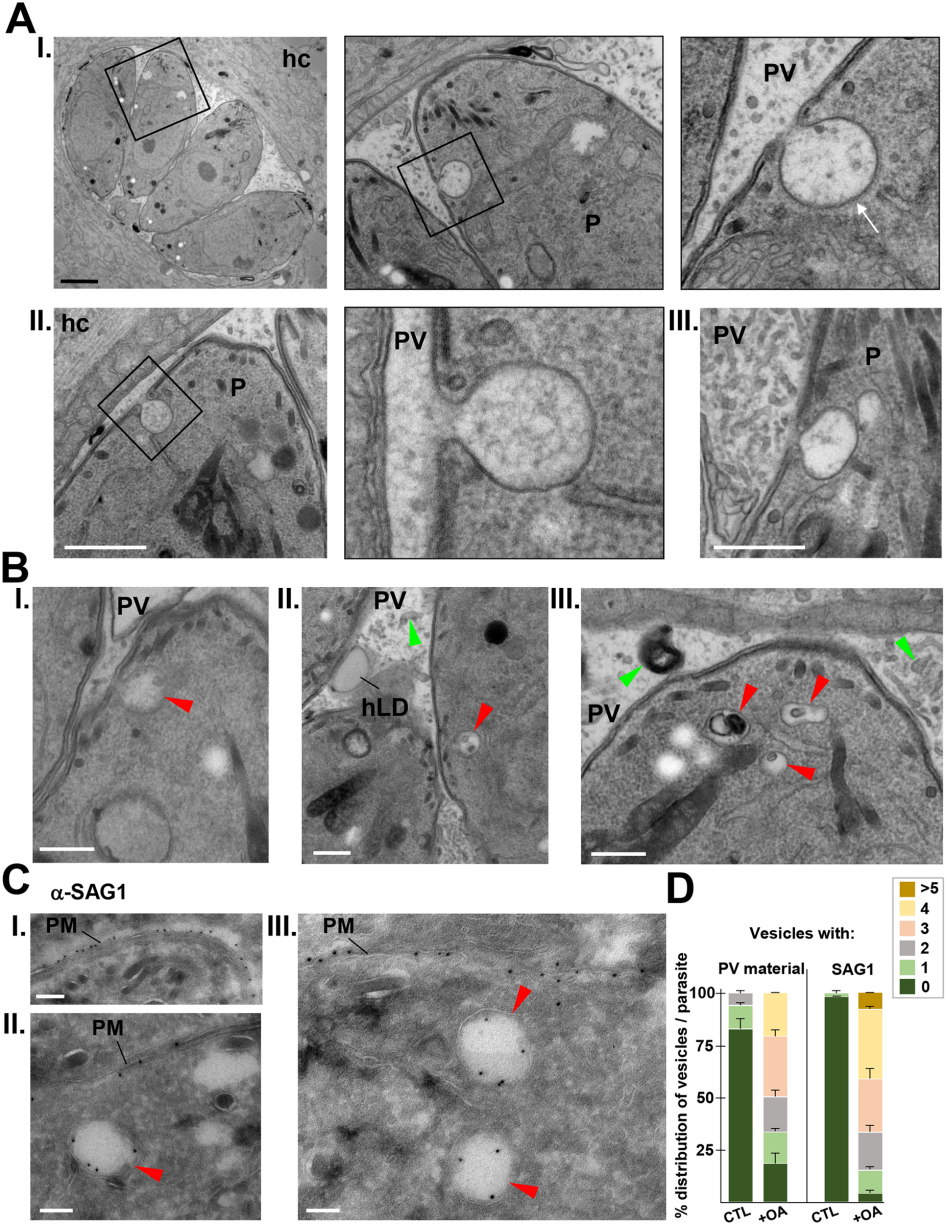
Ultrastructural detection of endocytic-like structures in *Toxoplasma*. **A**-**B.** Transmission EM of parasites in HFF incubated with 0.2 mM OA for 24 h. In A, panels I-III show three plasma membrane invaginations decorated with a cytoplasmic coat (arrow in I) and whose content displays resemblance with the material present in the PV lumen, suggestive of endocytic events. P, parasite; hc, host cell. Scale bars, 0.3 μm. In B, panels I-III illustrate intraparasitic vesicles containing osmiophilic material similar to that present in the PV lumen close to the parasite (red arrowheads). **C.** ImmunoEM for SAG1 detection, on parasites in HFF incubated with 0.2 mM OA for 24 h for SAG1 detection. Anti-SAG1-gold particles were observed on the limiting membrane of intracytoplasmic vesicles (red arrowheads). This antibody recognizes SAG1 epitopes on the extracellular moiety of SAG1 (panel I), making the SAG1 staining distributed on the internal leaflet of the organellar membrane, as expected for an endocytic event (panels II and III). PM, parasite plasma membrane. **D.** Quantification of intracytoplasmic vesicles either containing PV material or SAG1 staining in control and OA-loaded parasites. Data are means ± SD of 65 to 80 PV observed. All scale bars in A-C, 0.1 μm.

Regarding endocytic mechanisms for macromolecule acquisition, *Toxoplasma* is an intriguing organism since no internalization gates have been identified so far for this organism. Thus, the question remains as to whether *T*. *gondii* is able to incorporate large extracellular material from the PV lumen into an internal membrane system. Surprisingly, in our in-depth scrutiny of the membranous structures of *Toxoplasma* following exposure to excess OA (0.2 mM), we noticed one very large invagination of the parasite’s plasma membrane. This invaginated structure had a narrow neck and a diameter of, on average, 250 nm and was observed on many parasites (Fig 11A, panels I to III, corresponding to 3 different parasites; S7 Fig). This open structure was usually located at the anterior pole of the parasite, close to the apex, and was never present on dividing parasites. This invagination was clearly distinct from the micropore, a small static cup-shaped 20-nm pit of the plasma membrane [74], by its larger size and more post-nuclear localization. In addition, the structure and dimensions of the micropore remained unchanged upon OA addition, ruling out the possibility of micropore enlargement and transformation into this invagination (S8 Fig). The content of the invaginations was identical to the PV milieu, indicating a connection between these pits and the PV lumen. The cytoplasmic surface of these invaginations was also coated with a radiating, bristle-like structure (Fig 11A, arrow in panel I), reminiscent of the coat of specialized invaginations involved in endocytosis, (e.g., clathrin-coated pits or caveolae), indicating that these invaginations are not exocytic cups.

We also identified several cytoplasmic organelles containing material (e.g., membrane whorls) morphologically similar to the material present in the PV (Fig 11B, green arrowheads in panels I-III), which would be suggestive of endocytic vesicles (Fig 11B, red arrowheads in panels I-III). To provide further evidence towards the potential presence of an endocytic network within *Toxoplasma*, we performed an immunogold staining on intracellular *Toxoplasma* using antibodies against SAG1, a plasma membrane marker (Fig 11C, panel I). This would allow us to determine whether the cytoplasmic vesicles containing PV material detected in the parasite are derived from the plasma membrane. Several intracellular vesicles containing SAG1 on their limiting membrane were observed in parasites incubated with OA (Fig 11C, panels II and III). These SAG1-positive vesicles cannot correspond to exocytic vesicles due to their excessive size and their absence within parasites grown under normal (feeding) conditions. Quantitative analysis of cytoplasmic vesicles either containing PV material or SAG1 on their membrane reveal a dramatic increase in the number of these vesicles in OA-loaded vs. control parasites (Fig 11D). In fact, more than 96% of control parasites did not show any intracytoplasmic SAG1-positive structures. These observations may represent the first evidence of an endocytic event associated with an intravesicular system within *Toxoplasma*. Despite these cytoplasmic vesicles being predominantly detected under conditions of lipid gavage, they may be physiologically relevant and potentially involved in nutrient uptake and delivery to the parasite’s cytoplasm.

## Discussion

*Toxoplasma gondii* needs to salvage many essential metabolites, including lipids, from host cells to replicate and produce infectious progeny [75]. This parasite has evolved unique strategies to access the content of host organelles, which represent potential reservoirs of nutrients. For example, *Toxoplasma* intercepts host vesicular transport pathways, e.g., endo-lysosomes filled with LDL-cholesterol and Golgi Rab vesicles loaded with sphingolipids, and retrieves their lipid cargo [21, 24, 76]. Among lipid-enriched structures present in mammalian cells, lipid droplets (LD) represent an opportune source of various neutral lipids for any lipid scavengers. In this study, we demonstrated that *Toxoplasma* exploits host LD by relocating them to the PV and salvaging neutral lipids stored in these structures. Along with the exploitation of cytokines and lipoxins present in host LD as previously shown [9, 25, 26, 77], *Toxoplasma* also targets host LD for their neutral lipid stores. Additionally, we provide evidence that host LD are important for parasite infectivity: first, *Toxoplasma* growth is reduced in mutant MEF largely depleted of LD via the genetic ablation of *DGAT1* and *DGAT2* (D1D2KO); and second, parasites repetitively cultivated in D1D2KO cells, partially regain their growth capability when transferred to WT MEF.

During a *Toxoplasma* infection, the number of LD in the host cell fluctuates. Initially, the host LD population progressively increases, with a peak coincident with the first round of parasite replication (~8 h post-invasion). At this time, the global distribution of host LD is also prominently modified as many host LD cluster around the PV, and the transcription of host *DGAT2* is concomitantly upregulated, suggesting a stimulation of TAG synthesis in LD. After that period of time, the host LD number declines and *hsDGAT2* expression decreases but the vast majority of host LD remain accumulated around the PV. These changes in the cycle of host LD (biogenesis vs. breakdown for lipolysis), the distribution of host LD and the expression levels of *DGAT2* on LD reflect a highly dynamic status of host neutral lipid metabolism during infection. These significant changes may be driven by *Toxoplasma* ensuring the parasite’s survival and replication (e.g., modulation of immune processes and nutrient acquisition), but they could also be part of a host cell defense mechanism in response to parasite-mediated lipid imbalances as LD are uniquely responsive to changes in lipid homeostasis. How these processes are accomplished awaits further investigation.

Host LD overwhelmingly concentrate around the PV of *Toxoplasma*, a phenomenon that is amplified upon excess OA. The location of host LD near the PV membrane may benefit the parasite for the specific lipid content of these structures. For example, host LD that contain large amounts of esterified cholesterol, may act as an alternative source of cholesterol for *T*. *gondii* to endocytic organelles [21]. In fact, the parasite genome contains a potential lipid esterase (www.toxodb.org; TGME49_249350), that may de-esterify CE obtained from host LD to liberate cholesterol. In a similar manner, mammalian LD interact with many organelles for the exchange of materials [78]. LD-ER contact sites serve as conduits for the transport of phospholipids and proteins/enzymes with metabolic or signaling functions on LD. Trafficking of fatty acids also occurs from LD to mitochondria and peroxisomes, for catabolism through β-oxidation, and bidirectional phospholipid transport exists between LD and mitochondria. During lipophagy, physical contacts between LD and lysosomes occur, exemplified by the transfer of Rab7 to LD; by analogy, in the presence of excess OA, we observed an increase in the percent of PV containing Rab7 foci, with some foci corresponding to LD harboring Rab7 at their surface.

*Toxoplasma* may attract and retain host LD to its PV not just for their lipid content but also to take advantage of the enzymatic activities associated with LD. To this point, the parasite is sensitive to the inhibition of host ATGL activity via Atglistatin treatment [44] in a dose-dependent manner. This implies that the parasite may be affected by disruptions in the release of fatty acids and/or DAG from host LD. The parasite lacks an ATGL homolog, making host ATGL the sole target of Atglistatin, however, unintended effects of Atglistatin on off-target molecules in the parasite cannot be excluded. Further investigations would be required to ascertain whether *Toxoplasma* benefits from the local ATGL-mediated lipolysis of TAG occurring at the LD surface and salvages free fatty acids liberated from the host LD. Free fatty acids liberated from LD can be transported to the mitochondria for complete oxidation, resulting in ATP production. As *Toxoplasma* attracts both host LD and mitochondria to its PV, it is plausible that the parasite may then take advantage of products generated in the fatty acid β-oxidation cascade occurring in the host mitochondria. In support to this hypothesis is the parasite growth reduction in cells impaired in fatty acyl-CoA transfer to carnitine after exposure to Etomoxir [47] and in cells defective in acetyl-CoA generation from fatty acid oxidation after treatment with Trimetazidine [48]. A key aspect in the process of β-oxidation, which feeds the TCA cycle, is the transport of free fatty acids into the mitochondrion. However, the genome of *Toxoplasma* lacks homologs of carnitine palmitoyltransferases. In addition, no genes involved in the β-oxidation process could be identified in the *Toxoplasma* genome (e.g., acyl-CoA synthetases, mitochondrial fatty acid binding proteins, FAD-dependent acyl-CoA dehydrogenases, 3-ketoacyl coenzyme A thiolase), thus making the parasite unable to produce ATP via this pathway. These genomic analyses are further supported by metabolic studies showing that glucose and glutamine, and not fatty acids, are the only sources of precursors for the TCA cycle in *Toxoplasma*, and branched-chain ketoacid dehydrogenase, an enzyme localized to the mitochondrion, is likely the source of acetyl-CoA [79]. Further investigations using Type II strains of *Toxoplasma* that do not recruit host mitochondria to the PV, may provide insight into a potential functional coupling of host LD and mitochondria.

Finally, LD within activated macrophages, e.g., in the context of metabolic diseases or bacterial and parasitic infections, associate with phagosomes and discharge their content, e.g., arachidonate, into the phagosomal lumen [80, 81]. Many intracellular pathogens (e.g., *Trypanosoma cruzi*, *Leishmania amazonensis*, *Mycobacteria*, *Chlamydia trachomatis*) attract host LD to their phagosomal compartment and engulf them intact, by unknown mechanisms. For *C*. *trachomatis*, at least one molecular player involved in host LD recognition has been identified: the inclusion membrane protein IncA that binds to host ADRP at the LD surface [15]. Although the PV of *Toxoplasma* does not share any characteristics with a phagosome, *Toxoplasma* also internalizes host LD into the PV lumen, suggesting a common tactic shared by several intravacuolar pathogens to exploit LD cargoes.

The detection of Rab7-associated LD around and within the PV of *T*. *gondii* complements our previous study that has highlighted that the parasite is able to divert host Rab-labeled vesicles from the Golgi apparatus and sequester them into the PV lumen to retrieve their sphingolipid content [24]. This suggests that the parasite expresses proteins at the PV membrane that interact with host Rab proteins or their effectors. The re-routing and trapping of host LD inside the PV may in part explain the decline in the host LD number from 24 h p.i. as these structures may be consumed by the parasite. In fact, this hypothesis is supported by morphological evidence illustrating host LD often wrapped by the tubules of the IVN, a vacuolar network proposed to be involved in nutrient uptake. The IVN is initially generated from proteins and lipids secreted by the parasite and is subsequently modified by lipids scavenged from the host cell [57, 58]. The functionality of the IVN in nutrient accessibility for the parasites was demonstrated by the decreased ability of Δ*gra2*Δ*gra*6 parasites to salvage lipids from host LD. The PV lumen of these mutant parasites is indeed largely devoid of vesicles derived from the host cell (manuscript in preparation, JDR, SJN, KE, CP, EH, RH and IC). Of importance, the Δ*gra2* mutant is less virulent in animals, ascertaining an important role for the IVN in *Toxoplasma* infectivity [82]. We previously characterized a lipase, TgLCAT, which exhibits strong phospholipase A_2_ activity leading to the production of lysophospholipids that disrupt membranes, and localizes to the tubules of the IVN [59]. This enzyme may help in degrading the lipid monolayer of the host LD trapped inside the PV, resulting in the release of neutral lipids available for the parasite. This assumption is strongly supported by a reduced association lipids originating from host LD with Δ*lcat* mutant parasites.

Under conditions that stimulate host LD biogenesis, such as the exogenous addition of excess OA, *Toxoplasma* continuously salvages OA and/or lipids derived from the host cell’s metabolism of OA, resulting in an overaccumulation of various neutral lipid species. Similarly to mammalian cells, the parasite diverts excess lipids to neutral lipids in LD as an initial protective response against lipotoxic stress [83]. In response to excess OA, *Toxoplasma* upregulates a gene encoding DGAT, but unlike mammalian cells, it also increases transcriptional activities of cholesterol esterifying genes (*TgACAT2*); previously, *Toxoplasma* was shown to activate its ACAT genes also upon excess cholesterol in the medium [22]. Thus, efficient storage capacities for the prevention of lipotoxicity are essential for *Toxoplasma* survival. To this point, we previously demonstrated that a *T*. *gondii* strain lacking the *DGAT* gene was not viable, and parasites lacking either *ACAT* gene had severe growth defects whereas a double ACAT deletion was lethal [22, 23, 63]. Moreover, under conditions of lipid depletion (e.g., in cholesterol-free medium), the parasite relies on its cholesteryl ester store to survive [21], further underscoring the essentiality of neutral lipid stores for parasite development and infectivity. As *Toxoplasma* infects many cell types in the mammalian host, all of which vary in their lipid stores (e.g., abundant in intestinal and skeletal muscle; scarce in neurons [7]), it must adapt to these heterogeneous environments by modulating genes involved in lipid uptake and storage.

Concomitant with increased *TgDGAT* expression, the parasite upregulates an SCD gene orthologue coding for a lipogenic enzyme in part of a de novo fatty acid biosynthetic pathway, further increasing TAG production. The massive accumulation of LD, mainly filled with TAG and CE, in *Toxoplasma* in the presence of excess OA is suggestive of both an uncontrolled uptake of exogenous lipids by the parasite and its unrestricted capacity in storing surplus lipids in LD which occupy a large volume in the parasite’s cytosol. Similarly, adipocytes, which have evolved to stockpile lipids and energy in times of nutrient abundance, also upregulate SCD in the presence of excess lipids and form large size LD [69]. Lipotoxicity occurs, in pathologic states such as obesity, when the storage capacity for lipids is exceeded, leading to the hydrolysis of TAG pools and the increase of cellular concentrations of free fatty acids and other toxic lipids. In various animal models of obesity, SCD activity is abnormally elevated in the adipose tissue [69]. Most of non-adipocyte cells exposed to OA concentrations up to 0.5 mM are able to control the influx of fatty acids by storing TAG in LD and regulating TAG lipolysis and free fatty acid re-esterification. The parasite’s apparent gluttony for host OA and OA-derived lipids may place it at risk for lipotoxicity if its lipid storage threshold is reached at OA concentrations lower than 0.5 mM.

During our investigations into the morphology and membrane structure of *Toxoplasma* upon exposure to excess OA, we observed a large, well-structured invagination at the parasite’s plasma membrane, always located at the anterior pole of the parasite. One possible role for this structure would be its involvement in endocytosis. *T*. *gondii* has a dynamic acidified organelle, named VAC that contains cathepsin proteases implicated in protein degradation [84]. Strangely, no endocytic pit has been morphologically identified in *T*. *gondii*. The genome of *Toxoplasma* contains few homologues coding for proteins involved in an endocytic machinery, including AP2 complex, and the light and heavy chains of clathrin, but the parasite neither expresses these proteins at the plasma membrane nor forms clathrin-coated pits [85]. No caveolin homologues are present in the parasite genome, precluding also the existence of caveolae pits at the plasma membrane. However, the invagination detected upon excess OA incubation, which was distinct from the static micropore [74], is decorated with a regular cytoplasmic coat, morphologically reminiscent of mammalian clathrin-coated pits involved in receptor-mediated endocytosis. In addition, our EM images illustrate the presence of several cytoplasmic vesicles filled with heterogeneous material morphologically similar to that visible in the PV lumen. Moreover, the intracytoplasmic vesicles contain SAG1 on their limiting membrane, suggesting that the parasite is endowed with endocytic capability to internalize material from the PV lumen into a membranous network derived from its plasma membrane. These vesicles are unlikely not involved in exocytosis due to their excessive size and their occurrence in parasites only upon excess lipid availability. Additionally, the sole detection of these structures upon addition of 0.2 mM OA suggests that endocytic processes may be particularly fast in this parasite,thus impeding the trackability of these endocytic structures under normal (feeding) conditions. Further investigation on these novel structures, including the identification of the molecular components may provide additional information on their function. If these structures are indeed involved in endocytosis, it would divulge how the parasite internalizes and digests material, including host lipids, from the cellular environment.

## Materials and methods

### Ethics statement

No animals have been used in this study.

### Reagents and antibodies

All chemicals were obtained from Sigma (St Louis, MO) or Fisher (Waltham, MA) unless otherwise stated. [9,10-^3^H]oleic acid (sp act: 50 Ci/mmol) was purchased from Moravek (Brea, CA), [^3^H]uracil from PerkinElmer (Shelton, CT), 5-Butyl-4,4-Difluoro-4-Bora-3a,4a-Diaza-*s*-Indacene-3-Nonanoic Acid (C4-BODIPY-C9) from ThermoFisher Scientific (Waltham, MA) and 4,4-Difluoro-1,3,5,7,8-Pentamethyl-4-Bora-3a,4a-Diaza-*s*-Indacene (BODIPY 493/503) from Life Technologies (Carlsbad, CA). Non-polar lipid mixture for thin-layer chromatography was purchased from Matreya LLC (State College, PA). Atglistatin was purchased from Cayman Chemical (Ann Arbor, MI). The primary antibodies include: rabbit polyclonal anti-GRA7 [34], the rabbit polyclonal anti-aldolase and mouse monoclonal anti-Hsp70 (from Fidel Zavala, Johns Hopkins university, Baltimore), and the mouse monoclonal anti-SAG1 (from Jean-Francois Dubremetz, University of Montpellier, France). Secondary antibodies used for immunofluorescence were conjugated to Alexa^488^, Alexa^594^ or Alexa^350^ (Invitrogen, Carlsbad, CA). To prepare oleic acid (OA)-albumin complexes, sodium oleate was dissolved in H_2_O at a concentration of 100 mM, then thoroughly mixed by vortexing for 3 min with 5% Fatty Acid Free BSA to reach a final concentration of 10 mM OA-BSA complexes.

### Cell lines and culture conditions

Human foreskin fibroblasts (HFF) and HeLa cells were obtained from the American Type Culture Collection (Manassas, VA). GFP-ADRP HeLa cells were kindly provided by Raphael Valdivia (Duke University, North Carolina, USA; originally made by P. Targett-Adams and J. McLauchlan at the Medical Research Council Virology Unit, Institute of Virology, Glasgow, UK; [86]). Bone marrow-derived macrophages (BMDM) isolated from mice as described [87] was generously provided by Arturo Casadevall (Johns Hopkins University, Baltimore, MD). Wildtype mouse embryonic fibroblasts (MEF) and MEF lacking both *DGAT1* and *DGAT2* (D1D2KO) generously provided by Robert Farese (Harvard University, MA) were grown in D- minimum essential medium (MEM). All other cell lines were grown as monolayers and cultivated in α-MEM supplemented with 10% fetal bovine serum (FBS), 2 mM glutamine and penicillin/streptomycin (100 units/ml per 100 μg/ml), and maintained at 37°C in 5% CO_2_.

### Parasite cultivation and growth

The tachyzoites from the RH strain (type I lineage) were used throughout this study. *Toxoplasma* stably expressing RFP was kindly provided by Florence Dzierszinski (McGill University, Montreal, Canada). The Δ*gra2*Δ*gra6* tachyzoites were provided by Marie-France Cesbron-Delauw (Université de Grenoble, France) [61]. The Δ*lcat* and Δ*lcat*::LCAT overexpressor parasites were generated previously in our laboratory [59]. The parasites were propagated *in vitro* by serial passage in monolayers of HFF [88]. Under specific treatments, *T*. *gondii* growth was assayed either by parasite enumeration per PV or [^3^H]uracil incorporation assays. In the latter, MEF or HFF were grown until confluency in 24-well plates prior to infection with 1x10^5^ parasites for 20 h at 37°C in D-MEM (for MEF) or α-MEM (for HFF). Cells were then incubated with 1 μCi of [^3^H]uracil for 4 h at 37°C and the samples were processed as described [88]. In one set of replication assays, parasites were passaged in D1D2KO MEF for up to 12 serial passages.

### Real-time quantitative reverse transcription-PCR analysis

Confluent HFF were infected with *Toxoplasma* for 30 minutes, followed by PBS washes to remove extracellular parasites, then incubated with 0.2 mM OA for various times. RNA was extracted using *RNeasy Minikit* (Qiagen, Valencia, CA), according to the manufacturer’s protocol. The concentration was determined on a NanoDrop 1000 Spectrophotometer (Thermo Scientific, Waltham, MA). Amplified cDNA of the RNA samples was made using SuperScript III First Strand kit (Invitrogen). 100 ng of cDNA per sample was incubated with 2.25 μl each of forward and reverse appropriate primer and 17.5 μl of PowerUp SyBr Green Master Mix (Applied Biosystems) and run on StepOne Plus Real-Time PCR Systems (Applied Biosystems). The data of *TgACAT1*, *TgACAT2* and *TgDGAT* was normalized to the TgGT1 housekeeping gene whereas the data of *hsACAT*, *hsDGAT* and *hsADRP* was normalized to *hsGAPDH*. ΔΔCC values were calculated using RNA from uninfected HFF as control to assess transcript levels of *hsACAT*, *hsDGAT* and *hsADRP* in mammalian cells. For *Toxoplasma* transcript level quantification, ΔΔCC values were calculated using RNA from infected HFF without OA as control. The following primers were used: *TgACAT1*: (F) CGA CAT CCT CAT TTT CTA CAT TCT C and (R) GTG ACT CCA CTT GTA TCT TTG CTG; *TgACAT2*: (F) ATG CAT TTT TGT ATT CTA GGT GCA and (R) GGA GAA GGA GAA GAG TTG CAA A; *TgDGAT*: (F) GGA AGT GTG CTA TCC CTT ACA C and (R) CTC CCT TAC CAA AGC CGA TAA T; *TgSCD*: (F) GCA TTC TGC TCT CGC TAT CT and (R) GTG AGC ATT CTT CAT CCG TTT C. *TgGT1*: (F) GGC TAT TTT GGC ACC TTT CA and (R) AAC GGG AAG ACA AAC CAC AG; *hsADRP*: (F) CAG TAG TCG TCA CAG CAT CTT and (R) GAT TGA GGA GAG ACT GCC TAT TC; *hsACAT*: (F) CGG GCT AAC TGA TGT CTA CAA T and (R) GCA TAA GCG TCC TGT TCA TTT C; *hsDGAT1*: (F) CTGCAG GAT TCT TTA TTC AGC TC and (R) CAT TGC TCA AGA TCA GCA TCA C; *hsDGAT2*: (F) GCT ACA GGT CAT CTC AGT GCT C and (R) GTG AAG TAG AGC ACA GCG ATG AG; *hsGAPDH*: (F) GGT GTG AAC CAT GAG AAG TAT GA and (R) GAG TCC TTC CAC GAT ACC AAA G.

### Quantitative analyses of host LD number and morphology

Enumeration of total host LD identified by staining with BODIPY 493/503 in cells infected at various time points was performed using the “Find Object” tool in Volocity software, and host LD average surface area and volume were calculated. If ≥70% of total host LD in the host cell were clustered around the PV, the PV was marked as positive, and plotted as such in a histogram.

### Uptake assays of LD fluorescent dyes

Infected HFF, pretreated with 0.4 mM OA or not, were incubated with either 40 μM of C4-BODIPY-C9 or BODIPY 493/503 for 24 h, thoroughly washed with PBS, then chased for 2 h in α-MEM medium. Cells with pre-loaded BODIPY-stained host LD or C4-BODIPY-C9-stained host LD were infected with *Toxoplasma* (WT, RFP-RH or mutant parasites) at the indicated times prior to fixation for immunostaining or live cell imaging. In some assays, 0.2 mM of OA was added to the medium during *Toxoplasma* infections to ensure host LD maintenance in cells. For the quantification of basal LD stores in the parasite, and the neutral lipid uptake from host cell LD by various *Toxoplasma* strains, Volocity software was used to measure the sum intensity of the BODIPY 493/503 signal in PV (n > 30 PV per biological replicate). The sum intensity was then divided by the number of parasites within the PV to attain an averaged LD volume per parasite, measured in μm^3^. All quantifications were done in biological triplicates, and the means of means were plotted.

### Uptake assays for [^3^H]oleic acid

[^3^H]oleic acid was mixed with 100 mM of OA in ethanol, evaporated by N_2_, resuspended in 100 μl DMSO, and mixed with 900 μl of 7% fatty acid free BSA to reach a final concentration of 10 mM [^3^H]OA. HFF were seeded in 24-well plates and incubated with 0.1 or 0.4 mM [^3^H]OA to induce the formation of host LD containing radioactive OA. Excess [^3^H]OA was thoroughly washed from the medium and the cells were infected with *Toxoplasma* for 24 h or 36 h. The calcium ionophore A23187 (20 μM) was added to the cells to chemically induce *Toxoplasma* egress. The parasites were purified from the host cell debris using size exclusion columns (PD-10 desalting columns, GE Healthcare), followed by 3 rounds of washes. The radioactivity specifically associated with the parasite fraction was determined by scintillation counting (Multipurpose Scintillation Counter, Beckman, Brea, CA) and cpm values were normalized to the protein concentration of the sample using a Biorad Protein Assay (Biorad, Hercules, CA). These assays were graphed according to the means of means of radioactivity in cpm per mg in Excel (Microsoft).

### Mammalian cell transfection with Rab constructs

The GFP-Rab7 was kindly provided by Craig Roy (Yale University, School of Medicine, New Haven, CT) and the mCherry Rab7 was a gift Anne Hamacher-Brady (Johns Hopkins Bloomberg School of Public Health, Baltimore, MD). The EGFP-Rab18 construct was acquired from the Plasmid Repository Addgene (plasmid #49550). HFF were used for transfections using the Amaxa nucleofector kit V according to the manufacturer’s protocol (Lonza, Basel, Switzerland). Cells were transfected with 2.5 μg of plasmid DNA and left to recover overnight prior to infection with *Toxoplasma* for 30 minutes at 37°C, cascade-washed with PBS to remove extracellular parasites, and incubated for 24 h at 37°C.

### Thin layer chromatography

Egressed parasites (< 40–72 h infection times) from cells incubated with 0, 0.2 or 0.4 mM OA were spun down at 280 x *g* then resuspended in 3 ml of PBS, followed by sequential passaging through 20-, 22- and 27-gauge syringe needles at room temperature. The parasites were purified from the host cell debris using PD-10 desalting columns and protein concentration was determined using the bicinchoninic acid assay [89]. Total lipids from the same amount of purified parasites between the 3 experimental conditions (5x10^7^ parasites) were extracted with chlorofom:methanol:water (10:10:3, v/v/v), dried under liquid N_2_, and resuspended in chloroform. Lipids were fractionated by thin layer chromatography in petroleum ether:diethyl ether:acetic acid (80:20:1, v/v/v) or hexane:diethyl ether:acetic acid (90:10:1, v/v/v) on TLC Silica Gel 60 plates (Merck, Gibbsburg, NJ) run with lipid standards, visualized with iodine vapor, and band intensity of TAG and CE were measured by densitometry.

### Fluorescence microscopy

Immunofluorescence assays (IFA) on cells fix with 4% formaldehyde (Polysciences, Warrington, PA) plus 0.02% glutaraldehyde in PBS and permeabilize with 0.3% TritonX-100 was performed as described previously [24]. Coverslips were mounted using ProLong Diamond Antifade Mountant (Life Technologies) to minimize bleaching during microscopy observations. Cells were viewed with a Nikon Eclipse 90i equipped with an oil-immersion plan Apo 100x NA 1.4 objective and a Hamamatsu GRCA-ER camera (Hamamatsu Photonics, Hamamatsu, Japan). Optical z-sections with 0.2-μm spacing were acquired using Volocity software (PerkinElmer, Waltham, MA). The images were deconvolved using an iterative restoration algorithm and the registry was corrected using Volocity software. The positive product of the differences of the mean (PDM) images were calculated using Volocity software, which was also used to adjust brightness levels, cropping and resizing of the images obtained. Images of PV shown are representative of at least 20 PV.

### Electron microscopy

For transmission EM, HFF cells infected with *Toxoplasma* RH strain for 24 h (with or without OA) were fixed in 2.5% glutaraldehyde (EM grade; Electron Microscopy Sciences, Hatfield, PA) in 0.1 M sodium cacodylate buffer (pH 7.4) for 1 h at room temperature, and processed as described [90] before examination with a Philips CM120 Electron Microscope (Eindhoven, the Netherlands) under 80 kV. In one set of *Toxoplasma*-infected cells, malachite green was added during the aldehyde fixation and the osmium post-fixation to stain lipid bodies [56]. For immunoelectron microscopy (immunoEM), *Toxoplasma*-infected cells were fixed in 4% paraformaldehyde (Electron Microscopy Sciences) in 0.25 M HEPES (pH 7.4) for 1 h at room temperature, then in 8% paraformaldehyde in the same buffer overnight at 4°C. They were infiltrated, frozen and sectioned as previously described [21]. The sections were immunolabeled with anti-SAG1 antibodies (1:50) in PBS/1% fish skin gelatin, then with anti-IgG antibodies, followed directly by 10 nm protein A-gold particles to detect SAG1.

### Statistical methods

Data were displayed in box plots using Kaleidagraph software (Synergy Software) or graphed in Excel (Microsoft) with standard deviations displayed. Whiskers of the box plots represent the upper and lower values excluding outliers, outliers are marked as open circles, and the line inside the box is the median value. Means and standard deviations were calculated from three independent experiments using Excel (Microsoft). *p* values were calculated using either student *t*-test or a Chi-squared test in Excel (Microsoft).

## Supporting information

S1 Fig Clustering of host LD around the PV in mouse macrophages and fibroblasts. **A-B**. Fluorescence microscopy of primary BMDM (A) or MEF infected with RFP-expressing *Toxoplasma* for 24 h. Host LD were identified by staining with BODIPY 493/503 (green) and (DAPI) (blue, nucleus). The parasite is immunostained with GRA7 in the MEF whereas *Toxoplasma*-RFP was used to infect the BMDM. Uninfected and infected BMDM were incubated under control conditions while uninfected and infected MEF were incubated in medium containing 0.2 mM OA. For both cell types and conditions, host LD gathered around each PV (arrowheads).
(PDF)

S2 Fig Absolute values in cpm for the uracil incorporation assays shown in Fig 3 before normalization with respective controls set as 100%. Means ± SD are shown.
(PDF)

S3 Fig Co-distribution of host Rab7 on BODIPY 493/503-stained LD in mammalian cells. Fluorescence microscopy of *Toxoplasma*-infected HFF expressing mCherry-Rab7. Infected HFF expressing the mCherry Rab7 constructs were incubated in the absence or the presence of OA at 0.2 or 0.4 mM for 24 h, fixed and stained with BODIPY 493/503 and anti-GRA7 antibodies. Arrowheads show the PV. Extended focus images are shown for the BODIPY 493/503 (green), mCherry Rab7 (red) and the positive PDM, illustrating a subset of host LD colocalizing with mCherry-Rab7 more evidenced with added OA to the medium.
(PDF)

S4 Fig Detection of host Rab18-associated structures in the PV. **A.** Fluorescence microscopy of uninfected or 24 h-*Toxoplasma*-infected HFF expressing GFP-Rab18 grown without OA, with 0.2 or 0.4 mM OA. Coverslips were fixed and stained with antibodies for GRA7 (red; PV) and DAPI (blue; nucleus). Arrowheads pinpoint PV on phase images. The distribution of GFP-Rab18-positive vesicles (green) is shown in both uninfected and infected cells. Cropped images of the *Toxoplasma* PV are also shown in an optical XYZ slice to highlight the localization of host-derived GFP-Rab18 vesicles inside the PV of *Toxoplasma* (arrows). **B.** Quantification of the percentage of PV containing GFP-Rab18-associated structures within the lumen determined by XYZ visualization of Rab18 foci. Comparison was performed between PV in transfected HFF with or without OA as described in A. Data are values from one representative experiment done in triplicate biological samples (PV > 20 in each experiment). Chi-squared test, not significant between PV.
(PDF)

S5 Fig Ultrastructure of mammalian LD in the cytoplasm. **A**-**B.** Transmission EM of HFF incubated for 24 h with 0.2 mM showing the size and morphology of LD, with spherical (A) or crenelated shape (B). mt, mitochondrion. All scale bars, 0.5 μm.
(PDF)

S6 Fig Mammalian LD-related gene expression upon OA addition. Real-time PCR analysis of hsACAT, hsDGAT1, hsDGAT2, hsADRP and hsATGL gene expression in HFF in the absence (control) or the presence of 0.2 mM OA. Means ± SD of 3 assays in triplicates, showing significant increase of hsDGAT1, hsDGAT2 and hsADRP transcripts upon OA addition relative to control (*p <0.02; **p <0.03).
(PDF)

S7 Fig Detection of an invaginated pit at the apical end of *Toxoplasma*. Transmission EM of a PV of *Toxoplasma* cultivated in the presence of 0.2 mM OA for 24 h illustrating an invaginated pit (red circles) on two parasites. The pit was visible on all sections that were passing through the apex of the parasites. Scale bar, 0.5 μm.
(PDF)

S8 Fig Morphology of the micropore upon OA addition. **A-B**. Transmission EM of *Toxoplasma* cultivated in HFF under normal conditions (A) or in the presence of 0.2 mM OA for 24 h (B) showing no difference in micropore (red arrows or circles) size or morphology with OA added to the medium. All scale bars, 0.5 μm.
(PDF)
