# Supplementary figures and images for: Host lipid droplets: An important source of lipids salvaged by the intracellular parasite Toxoplasma gondii
Source: PLoS Pathog. 2017 Jun 1;13(6):e1006362. doi: 10.1371/journal.ppat.1006362 (PMC5469497; doi:10.1371/journal.ppat.1006362)

**Figure S1**

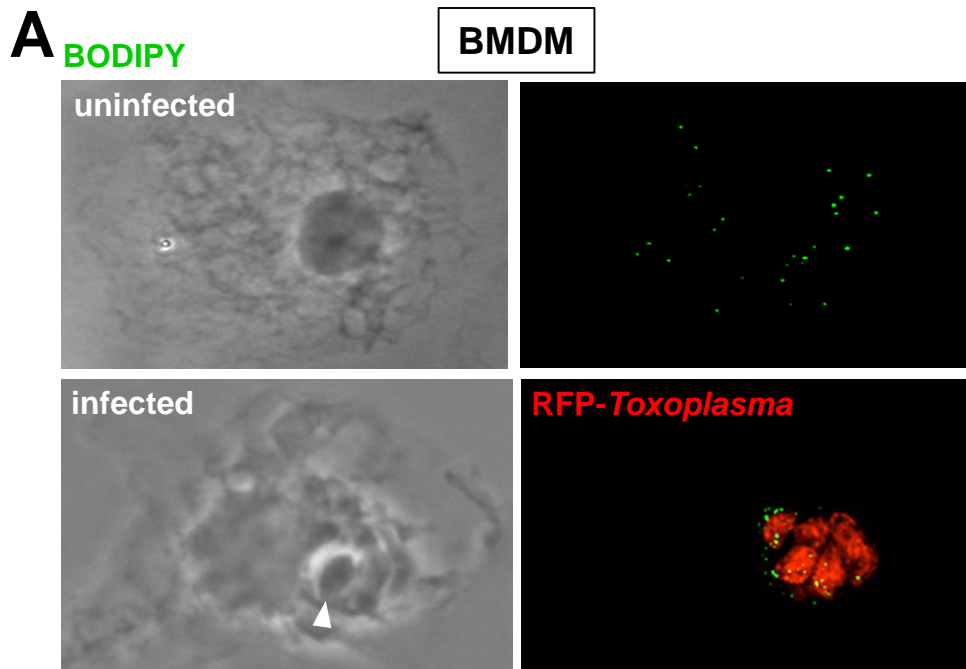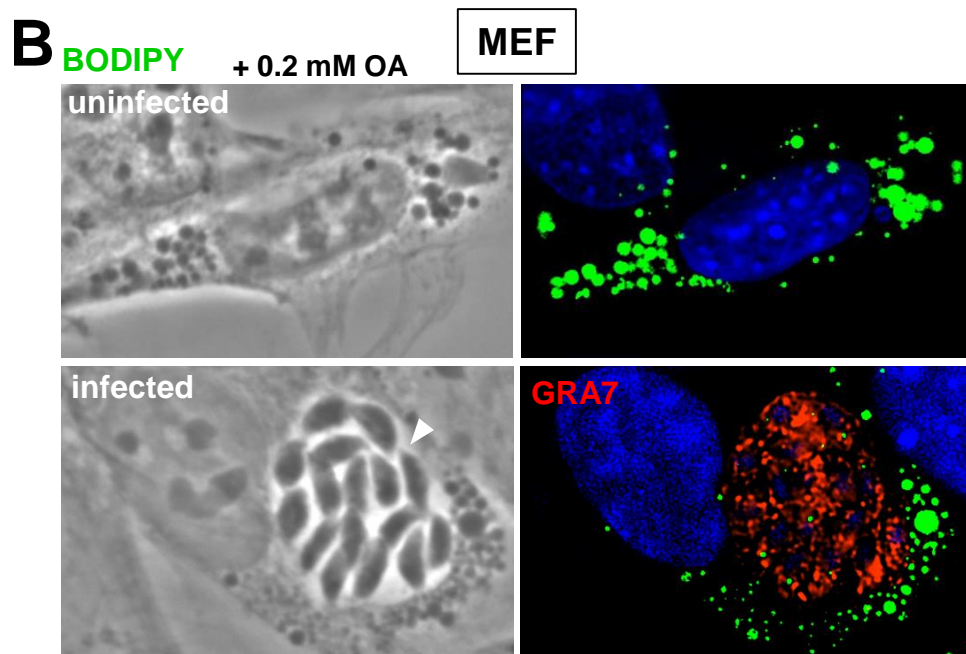

Supplement: S1 Fig — A-B. Fluorescence microscopy of primary BMDM (A) or MEF infected with RFP-expressing Toxoplasma for 24 h. Host LD were identified by staining with BODIPY 493/503 (green) and (DAPI) (blue, nucleus). The parasite is immunostained with GRA7 in the MEF whereas Toxoplasma-RFP was used to infect the BMDM. Uninfected and infected BMDM were incubated under control conditions while uninfected and infected MEF were incubated in medium containing 0.2 mM OA. For both cell types and conditions, host LD gathered around each PV (arrowheads). (PDF) [file ppat.1006362.s001.pdf]

**Figure S3**

mCh-Rab7  
BODIPY  
GRA7

+ 0.2 mM OA

+ 0.4 mM OA

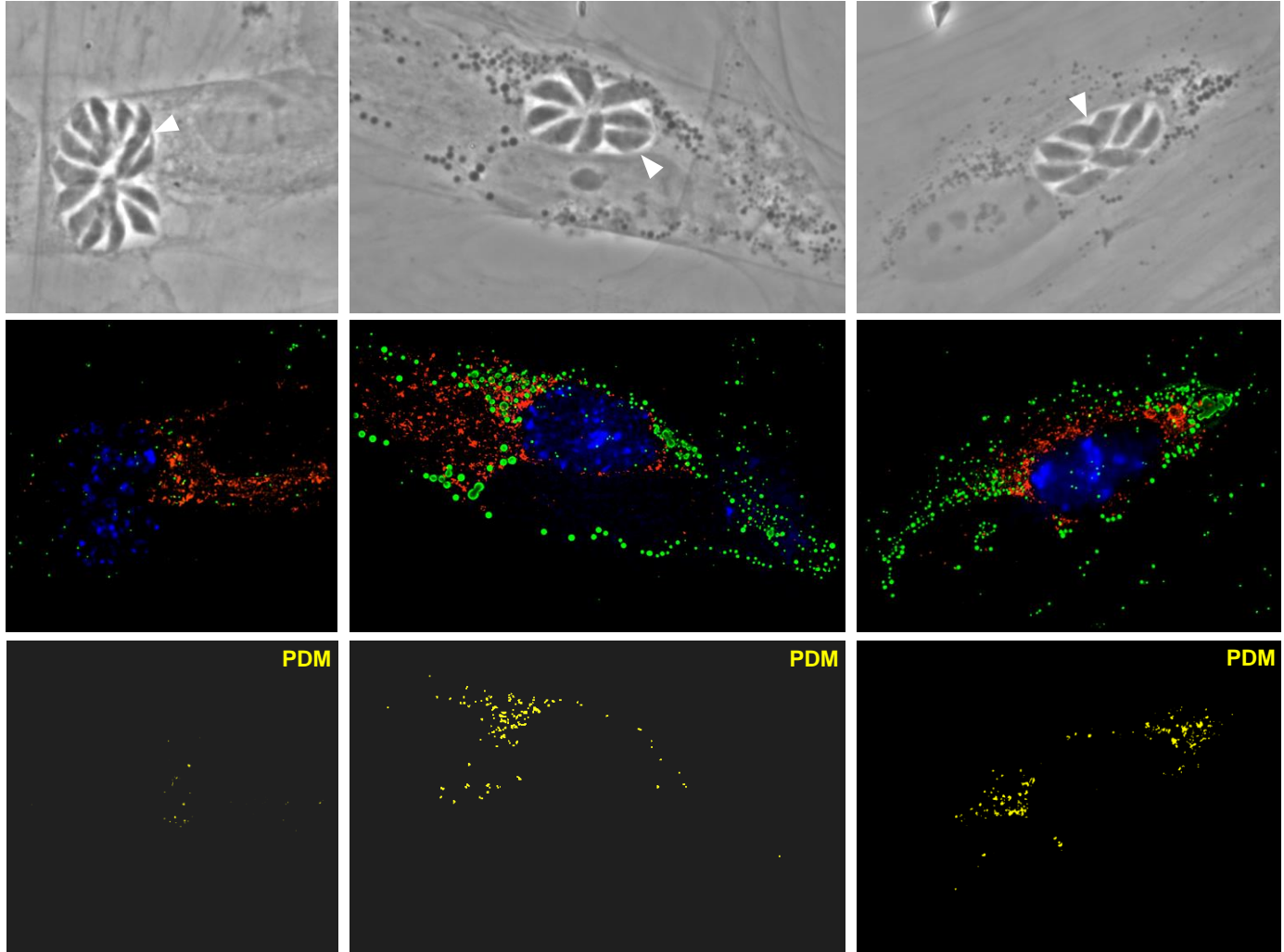

Supplement: S3 Fig — Fluorescence microscopy of Toxoplasma-infected HFF expressing mCherry-Rab7. Infected HFF expressing the mCherry Rab7 constructs were incubated in the absence or the presence of OA at 0.2 or 0.4 mM for 24 h, fixed and stained with BODIPY 493/503 and anti-GRA7 antibodies. Arrowheads show the PV. Extended focus images are shown for the BODIPY 493/503 (green), mCherry Rab7 (red) and the positive PDM, illustrating a subset of host LD colocalizing with mCherry-Rab7 more evidenced with added OA to the medium. (PDF) [file ppat.1006362.s003.pdf]

**Figure S4**

**A**

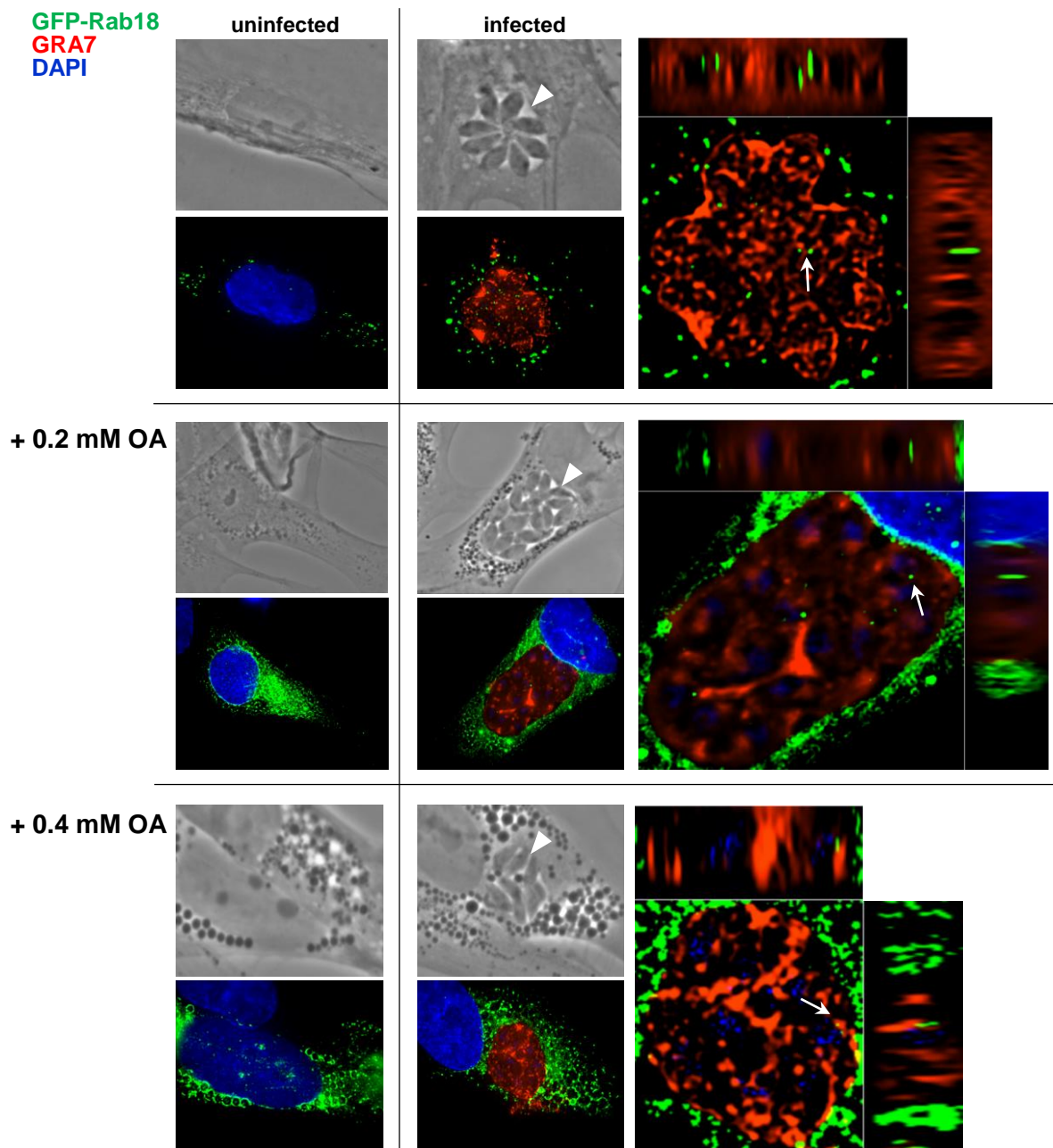

**B**

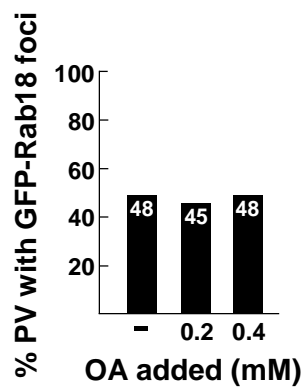

Supplement: S4 Fig — A. Fluorescence microscopy of uninfected or 24 h-Toxoplasma-infected HFF expressing GFP-Rab18 grown without OA, with 0.2 or 0.4 mM OA. Coverslips were fixed and stained with antibodies for GRA7 (red; PV) and DAPI (blue; nucleus). Arrowheads pinpoint PV on phase images. The distribution of GFP-Rab18-positive vesicles (green) is shown in both uninfected and infected cells. Cropped images of the Toxoplasma PV are also shown in an optical XYZ slice to highlight the localization of host-derived GFP-Rab18 vesicles inside the PV of Toxoplasma (arrows). B. Quantification of the percentage of PV containing GFP-Rab18-associated structures within the lumen determined by XYZ visualization of Rab18 foci. Comparison was performed between PV in transfected HFF with or without OA as described in A. Data are values from one representative experiment done in triplicate biological samples (PV > 20 in each experiment). Chi-squared test, not significant between PV. (PDF) [file ppat.1006362.s004.pdf]

**Figure S5**

**A**

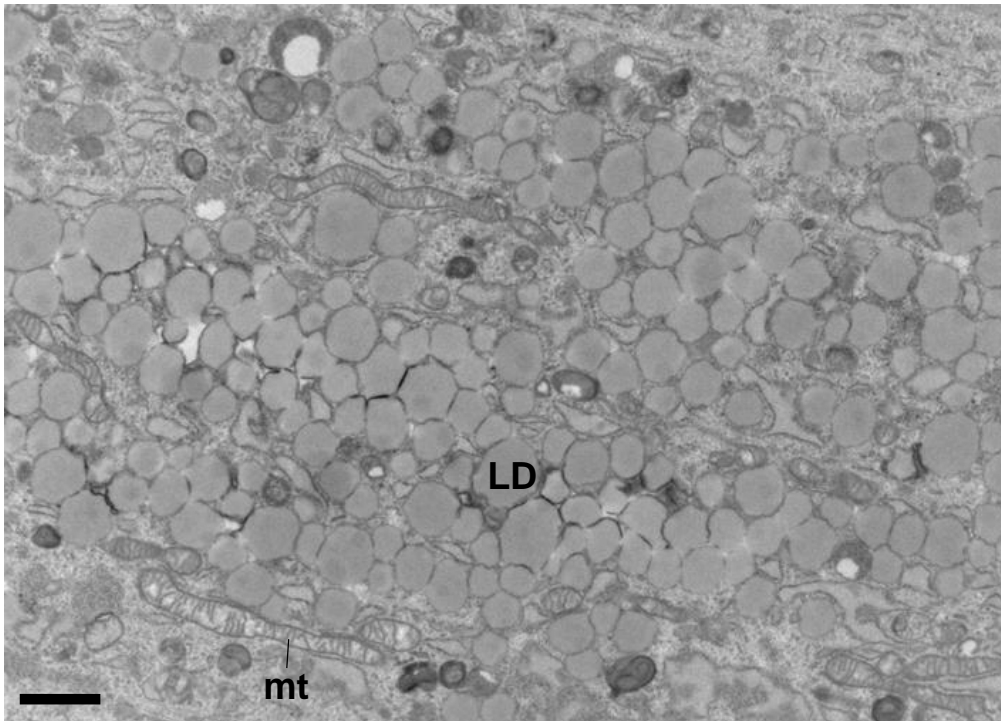

**B**

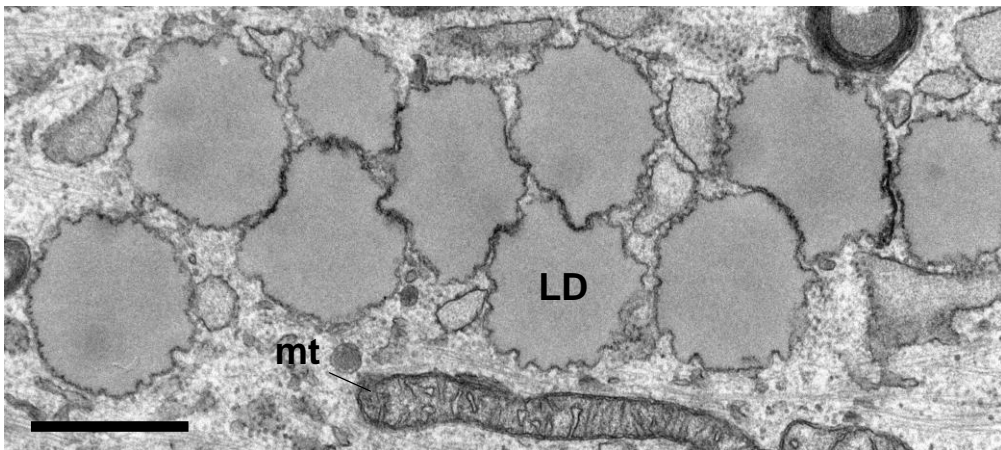

Supplement: S5 Fig — A-B. Transmission EM of HFF incubated for 24 h with 0.2 mM showing the size and morphology of LD, with spherical (A) or crenelated shape (B). mt, mitochondrion. All scale bars, 0.5 μm. (PDF) [file ppat.1006362.s005.pdf]

**Figure S6**

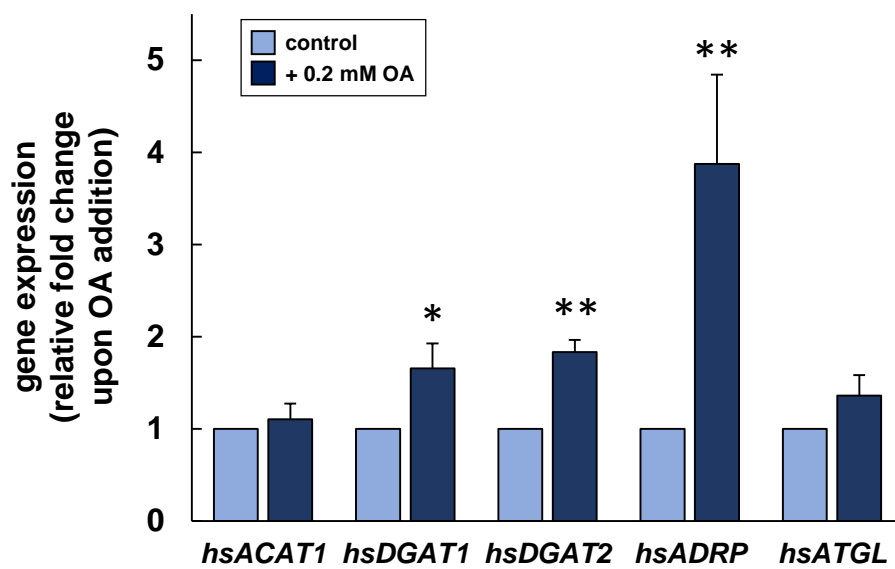

Supplement: S6 Fig — Real-time PCR analysis of hsACAT, hsDGAT1, hsDGAT2, hsADRP and hsATGL gene expression in HFF in the absence (control) or the presence of 0.2 mM OA. Means ± SD of 3 assays in triplicates, showing significant increase of hsDGAT1, hsDGAT2 and hsADRP transcripts upon OA addition relative to control (*p <0.02; **p <0.03). (PDF) [file ppat.1006362.s006.pdf]

**Figure S7**

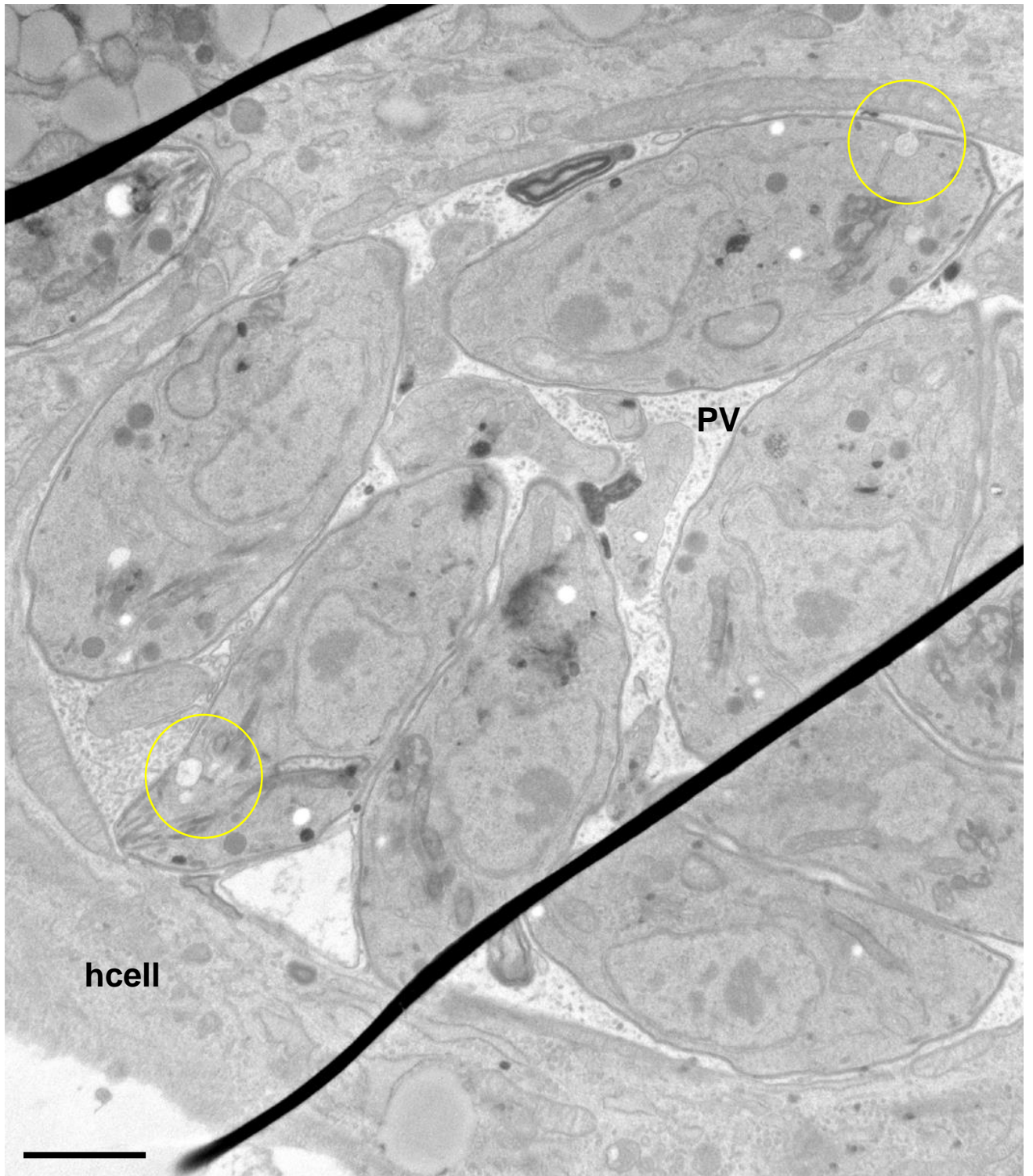

Supplement: S7 Fig — Transmission EM of a PV of Toxoplasma cultivated in the presence of 0.2 mM OA for 24 h illustrating an invaginated pit (red circles) on two parasites. The pit was visible on all sections that were passing through the apex of the parasites. Scale bar, 0.5 μm. (PDF) [file ppat.1006362.s007.pdf]

**Figure S8**

**A** control

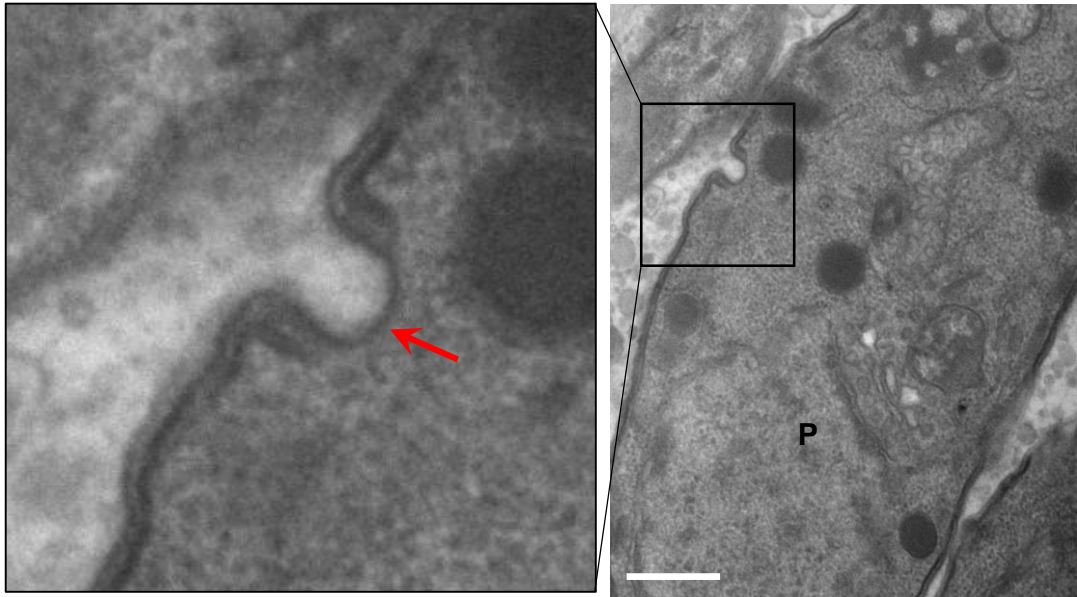

**B** + 0.2 mM OA

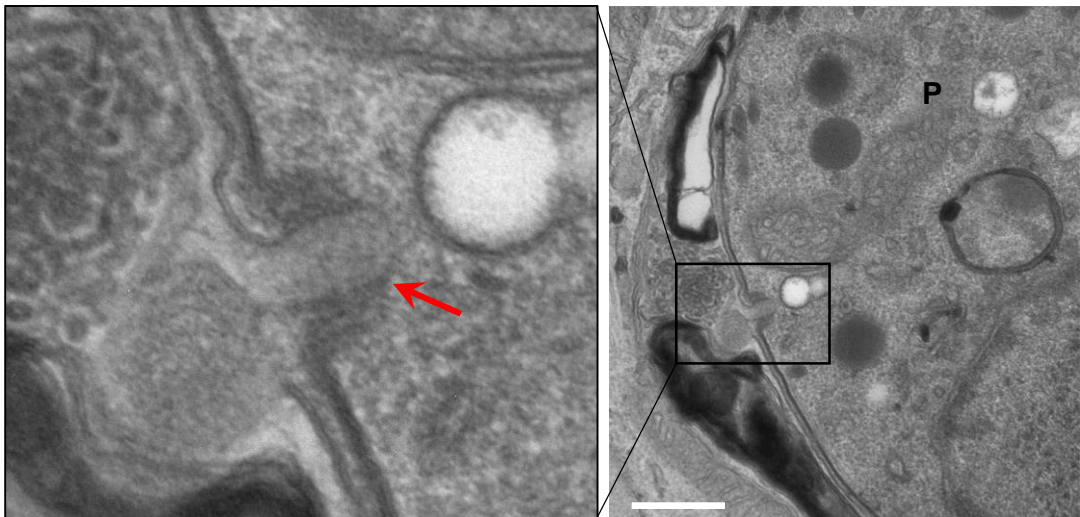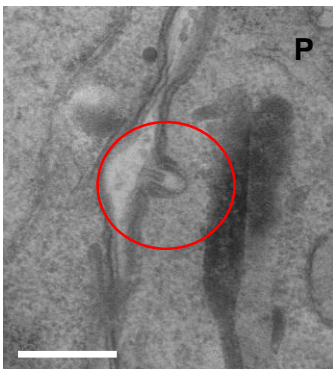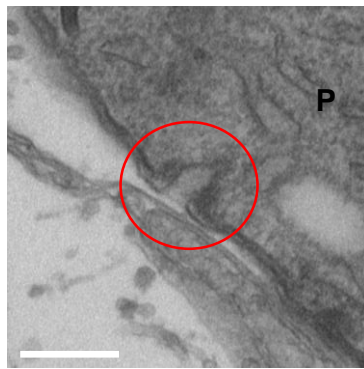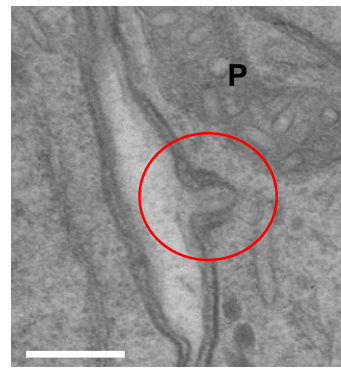

Supplement: S8 Fig — A-B. Transmission EM of Toxoplasma cultivated in HFF under normal conditions (A) or in the presence of 0.2 mM OA for 24 h (B) showing no difference in micropore (red arrows or circles) size or morphology with OA added to the medium. All scale bars, 0.5 μm. (PDF) [file ppat.1006362.s008.pdf]
