# Supplementary material for: Host lipid droplets: An important source of lipids salvaged by the intracellular parasite Toxoplasma gondii
Source: PLoS Pathog. 2017 Jun 1;13(6):e1006362. doi: 10.1371/journal.ppat.1006362 (PMC5469497; doi:10.1371/journal.ppat.1006362)
Supplement: S2 Fig — Means ± SD are shown. (PDF) [file ppat.1006362.s002.pdf]

Figure S2

For Figure 3B:

Exp. 1<br>130,818 ± 28,171 cpm (WT)<br>65,842 ± 5,043 cpm (D1D2KO)	***Exp. 2***<br>45,549 ± 2,387 cpm (WT)<br>29,520 ± 2,977 cpm (D1D2KO)
Exp. 3<br>52,700 ± 4,642 cpm (WT)<br>37,454 ± 2,840 cpm (D1D2KO)	***Exp. 4***<br>154,773 ± 39,422 cpm (WT)<br>65,842 ± 5,043 cpm (D1D2KO)

For Figure 3C:

Exp. 1 (P8)<br>26,583 ± 3,599 cpm (condition I)<br>15,775 ± 255 cpm (condition II)<br>11,578 ± 1,380 cpm (condition III)<br>8,775 ± 735 cpm (condition IV)	***Exp. 2 (P9)***<br>69,994 ± 5,213 cpm (condition I)<br>40,043 ± 12,048 cpm (condition II)<br>31,154 ± 1,943 cpm (condition III)<br>16,924 ± 8,618 cpm (condition IV)
Exp. 3 (P10)<br>66,996 ± 5,835 cpm (condition I)<br>61,901 ± 3,169 cpm (condition II)<br>54,214 ± 2,362 cpm (condition III)<br>48,646 ± 2,140 cpm (condition IV)	***Exp. 4 (P12)***<br>35,679 ± 9,340 cpm (condition I)<br>27,956 ± 804 cpm (condition II)<br>10,709 ± 667 cpm (condition III)<br>2,563 ± 114 cpm (condition IV)

For Figure 3D:

Atglistatin:	***Exp. 1***<br>99,495 ± 2,205 (DMSO)<br>78,268 ± 7,770 (25 µM)<br>60,477 ± 22,890 (50 µM)<br>29,058 ± 5,449 (100 µM)	***Exp. 2***<br>56,183 ± 1,567 (DMSO)<br>51,185 ± 2,000 (25 µM)<br>44,272 ± 6,170 (50 µM)<br>27,490 ± 2,297 (100 µM)	***Exp. 3***<br>138,204 ± 14,940 (DMSO)<br>122,916 ± 1,911 (25 µM)<br>112,190 ± 6,358 (50 µM)<br>85,316 ± 921 (100 µM)
Etomoxir:	***Exp. 1***<br>99,495 ± 2,205 (DMSO)<br>48,495 ± 12,657 (50 µM)<br>52,996 ± 11,311 (100 µM)<br>6,789 ± 296 (300 µM)	***Exp. 2***<br>56,183 ± 1,567 (DMSO)<br>43,775 ± 4,161 (50 µM)<br>21,783 ± 12,266 (100 µM)<br>8,905 ± 2,543 (300 µM)	***Exp. 3***<br>138,204 ± 14,940 (DMSO)<br>132,073 ± 3,011 (50 µM)<br>117,989 ± 4,622 (100 µM)<br>26,499 ± 1,266 (300 µM)
	Exp. 4<br>59,838 ± 10,977 (DMSO)<br>45,651 ± 3,973 (50 µM)<br>36,332 ± 4,975 (100 µM)<br>2,589 ± 1,785 (300 µM)		
Trimetazidine:	***Exp. 1***<br>50,307 ± 9,072 (PBS)<br>41,748 ± 7,727 (10 µM)<br>28,091 ± 4,921 (100 µM)<br>30,665 ± 7,012 (300 µM)	***Exp. 2***<br>32,754 ± 6,052 (PBS)<br>26,319 ± 702 (10 µM)<br>23,800 ± 1,730 (100 µM)<br>18,893 ± 2,284 (300 µM)	***Exp. 3***<br>66,708 ± 6,385 (PBS)<br>40,043 ± 14,756 (10 µM)<br>31,154 ± 2,380 (100 µM)<br>16,924 ± 10,555 (300 µM)
